# Mutual Regulation of ncRNAs and Chromatin Remodeling Complexes in Normal and Pathological Conditions

**DOI:** 10.3390/ijms24097848

**Published:** 2023-04-25

**Authors:** Irina V. Bure, Marina V. Nemtsova

**Affiliations:** 1 Laboratory of Medical Genetics, I.M. Sechenov First Moscow State Medical University, 119991 Moscow, Russia; nemtsova_m_v@mail.ru; 2 Laboratory of Epigenetics, Research Centre for Medical Genetics, 115522 Moscow, Russia

**Keywords:** chromatin remodeling complexes, long non-coding RNA, microRNA, epigenetic regulation, cancer, epi-drugs

## Abstract

Chromatin remodeling is the one of the main epigenetic mechanisms of gene expression regulation both in normal cells and in pathological conditions. In recent years, a growing number of investigations have confirmed that epigenetic regulators are tightly connected and form a comprehensive network of regulatory pathways and feedback loops. Genes encoding protein subunits of chromatin remodeling complexes are often mutated and change their expression in diseases, as well as non-coding RNAs (ncRNAs). Moreover, different mechanisms of their mutual regulation have already been described. Further understanding of these processes may help apply their clinical potential for establishment of the diagnosis, prognosis, and treatment of the diseases. The therapeutic targeting of the chromatin structure has many limitations because of the complexity of its regulation, with the involvement of a large number of genes, proteins, non-coding transcripts, and other intermediary molecules. However, several successful strategies have been proposed to target subunits of chromatin remodeling complexes and genes encoding them, as well as the ncRNAs that regulate the operation of these complexes and direct them to the target gene regions. In our review, we focus on chromatin remodeling complexes and ncRNAs, their mutual regulation, role in cellular processes and potential clinical application.

## 1. Introduction

Chromatin remodeling is the one of the main epigenetic mechanisms of gene expression regulation, both in normal conditions and in various diseases. Nucleosomes are the basic elements of eukaryotic DNA structural organization. Each nucleosome consists of 146 bp DNA wrapped around a histone octamer, composed of two copies each of histones H2A, H2B, H3, and H4 [1]. Chromatin remodeling protein complexes, together with the other epigenetic mechanisms such as DNA methylation/demethylation and histone modifications, alter chromatin configuration from a tightly condensed state (heterochromatin), when genes are transcriptionally inactivated, to a loosened one (euchromatin) [2]. Using energy from ATP hydrolysis, chromatin remodeling complexes slide, eject, or modify nucleosomes, thereby affecting DNA accessibility [3,4]. Because of this ability, they regulate important cellular processes such as transcription, recombination, replication, and DNA repair, and their deregulation leads to serious pathologies [1].

Relatively recent confirmation of the non-protein-coding transcriptome functionality and further understanding of its involvement in the regulation of important biological processes led to the recognition of non-coding RNAs (ncRNAs) as epigenetic regulators. NcRNAs are numerous, diverse in forms and functions, and can interact with different molecules, including RNA, DNA, and proteins, both directly and indirectly [5]. The interplay of different classes of epigenetic regulators suggests the mutual regulation of ncRNAs and chromatin remodeling machinery. A number of recent investigations revealed a broad association of ncRNAs with chromatin remodeling complexes, supporting this suggestion [6]. Both subunits of chromatin remodeling complexes and ncRNAs were found to be mutated and/or deregulated in human diseases and affecting the expression program of many genes associated with their development and progression [7]. Currently, advances in genomic and proteomic techniques allow us to obtain a significant amount of information about the aberrations of epigenetic mechanisms and apply them practically. Targeting the epigenetic regulators involved in pathogenesis is an attractive option, despite not being sufficiently studied yet. As groups of epigenetic regulators, chromatin remodeling complexes and ncRNAs evoke reversible alterations in gene expression. Furthermore, their deregulation appears at the early stages of diseases and significantly correlates with their progression. These characteristics make them prominent diagnostic and prognostic biomarkers, as well as potential targets for therapeutic agents [8].

In our review, we summarize the current data of regulatory interactions between chromatin remodeling complexes and ncRNAs and discuss their role in pathologies, including cancer, as well as potential application for diagnostics and therapy.

## 2. Chromatin Remodelers in Normal and Pathological Conditions

Chromatin remodelers are classified into four families depending on their subunit composition and the mechanism of nucleosome manipulation. There are switch/sucrose non-fermentable complexes (SWI/SNF), imitation switch complexes (ISWI), chromodomain helicase DNA-binding complexes (CHD) and inositol-requiring 80 (INO80) complexes (INO80/SWR1). All of them are evolutionarily conserved and demonstrate high variability of subfamily members related to their different functions and tissue- and cell type-specificity [9].

All chromatin remodeling complexes include the catalytic subunit containing an SNF2-like ATPase domain, and one or several accessory subunits that determine a biological role and specificity of the chromatin remodeling complex [1]. Epigenetic modifications occur through binding of the complexes to specific chromatin domains using energy from ATP hydrolysis to disrupt the interactions between histones and DNA, thus altering the chromatin structure in a non-covalent manner [10].

Chromatin remodelers play an important role in the regulation of gene expression, DNA replication and repair, developmental processes, pluripotency, and chromosome segregation. Deregulation of chromatin remodeling leads to critical changes of these processes in the cell [11].

### 2.1. SWI/SNF Complexes

The SWI/SNF complex is the best characterized family of chromatin remodeling complexes. They act by recognizing the nucleosome and DNA with a high affinity, shifting, and exposing DNA segments along the surface of the nucleosome. Depending on the ATPase activity, it results in nucleosome sliding or in destabilizing and the removal of H2A-H2B dimers or entire histone cores [12]. Therefore, SWI/SNF complexes mediate nucleosome reorganization and regulate the access of transcription factors, allowing genes to be activated or repressed [13].

SWI/SNF complexes in human cells consist of 12–15 subunits and can be divided in three subclasses based in their core components: BRG/hBRM-associated factors (BAF) complexes, polybromo-associated BAF (PBAF) complexes, and non-canonical BAF (ncBAF) complexes [14]. They contain the mutually exclusive catalytic ATPase subunits SMARCA2 (SWI/SNF related, matrix associated, actin dependent regulator of chromatin, subfamily A, member 2), also called Brahma (BRM), SMARCA4 or BRM-related gene 1 (BRG1), and a number of other core components and accessory subunits [15]. In addition to either SMARCA4 or SMARCA2, BAF complexes consist of the exclusive subunits ARID1A or ARID1B (BAF250A/B), BRD9, and SS18. Exclusive subunits of PBAF complexes are SMARCA4, ARID2 (BAF200), PBRM1 (BAF180), PHF10, and BRD7 [16]. The other core components are the same for both BAF and PBAF subclasses and include SMARCC1 (BAF155), SMARCC2 (BAF170), and SMARCB1 (SNF5/BAF47/INI1) [7]. In ncBAF complexes, the central ARID1A subunit is replaced by the GLTSCR1. In addition, they have no SMARCC2, SMARCE1, or SMARCB1 subunits, but include the BRD9 subunit, which is not present in the other two groups of SWI/SNF complexes [14].

Because of the variability of subunits and structural diversity, SWI/SNF chromatin remodeling complexes interact with enhancers and promoter regions by cell-type specifically, and participate in numerous cellular processes, such as proliferation, differentiation, development, DNA repair, and immunity [17]. The role of SWI/SNF complexes in gene expression regulation enables their involvement in disease development and progression. Thus, mutations in SWI/SNF-encoding genes were found in approximately 20% of all cancers. There are mostly loss-of-function mutations, such as deleterious missense mutations, frameshift mutations, and chromosomal deletions [18]. For example, *ARID1A* is mutated in 46–57% of ovarian clear-cell carcinoma, 27% of gastric cancer, 17.5% of colon and rectal cancers, 16.7% of cholangiocarcinoma, 13% of hepatocellular carcinoma, 11% of colorectal adenocarcinoma, 9% of endometrial carcinoma, and 2.5% of malignant melanoma [19]. ARID1A is the most frequently mutated SWI/SNF subunit in different tumors, and cancer-related mutations are spread across almost all regions of the *ARID1A* gene [14]. Being downregulated, the tumor suppressor gene *ARID1A* promotes cancer development through perturbations in the DNA-damage response and by activation of the PI3K/AKT/mTOR cell-cycle pathway [20,21].

The gain-of-function mutations in SWI/SNF-encoding genes occur much more rarely. Among them are the genetic aberrations of SS18 by the fusion of the SS18 gene and the SSXs (SSX1, SSX2, or SSX4) that were detected in aggressive synovial sarcoma [22].

Dysregulation of SWI/SNF gene expression was also associated with cancer prognosis and response to treatment. The tumors that bear mutations in the *SMARCA4* gene are more aggressive and have been associated with a poorer prognosis [14]. The inactivation of SMARCA2 or SMARCA4 makes cancer cells more sensitive to cisplatin. Loss of SMARCA4 is associated with the resistance to topoisomerase II inhibitors and enhances sensitivity to docetaxel, whereas its low expression was described to increase platinum-based chemotherapy sensitivity [17].

The mutations of genes coding SWI/SNF complexes have been described also in other diseases. Thus, the mutations in *SMARCA2* can cause Nicolaides-Baraitser syndrome [23]. Mutations in the ATPase subunits of ARID1B are associated with the development of a mild form of the Coffin-Siris syndrome [24]. Classic and more severe Coffin-Siris syndromes are caused by mutations in *SMARCA4*, the common core subunit SMARCB1, and accessory subunits SMARCE1, ARID1A, and ARID2 [25]. Mutations in *SMARCB1* can also lead to the DOORS syndrome or the Kleefstra syndrome, depending on their location [11].

### 2.2. ISWI Complexes

ISWI complexes contain 2–4 subunits, including one of two conserved ATPase subunits: SMARCA5 (SNF2H) or SMARCA1 (SNF2L). They have a highly homologous amino acid sequence. However, being associated with different accessory subunits, they are expressed in a tissue-specific manner and have different functions [26]. SMARCA5 forms stable complexes CHRAC, ACF, WICH, NoRC, and RSF, whereas SMARCA1 was found only in CERF and NURF complexes [27].

ISWI complexes participate in the transformation of the initial histone–DNA complexes into mature canonical octameric nucleosomes and the spacing of nucleosomes at fixed distances [28]. They are involved in the regulation of transcription, recombination, and the DNA damage response [27].

Currently, a number of investigations have demonstrated the critical role of ISWI complexes in pathological processes, including tumorigenesis. The TCGA database has revealed abnormal expression of ISWI subunits in different types of cancer. The genetic abnormality is a main factor that may induce gain- or loss-of-function properties of ISWI subunits, thus affecting their interplay with transcription factors and gene regulatory networks. There are somatic mutations, translocations, and copy number changes [26]. In addition, some ISWI subunits are considered to be prognostic [26]. For example, the *BPTF* gene copy number is frequently amplified in tumors, such as lung cancer, neuroblastomas [29], and melanoma [30]. Furthermore, gain of the 17q24.3 locus was associated with poor prognosis in 67% of the BPTF-positive lung tumors [29]. Upregulation of *SMARCA5* in ovarian cancer and hepatocellular carcinoma contributes to tumor cell survival, proliferation, and growth [26]. It was also frequently overexpressed in acute myeloid leukemia [31] and breast cancer, where it was positively correlated with tumor size, TNM stage, and a poor overall survival [32].

### 2.3. CHD Complexes

The CHD family of chromatin remodeling complexes includes proteins with two tandem chromodomains that facilitate the binding to the methylated histone residues and two SNF2-like ATP-dependent helicase domains, promoting the mechanical disruption of DNA-histone contacts. Therefore, the core histones either slide along the DNA template or can be evacuated and deposited onto another DNA strand [33]. CHD complexes slide or eject nucleosomes to promote transcription or play a repressive role with histone deacetylases [7]. They are involved in stem cell maintenance, survival, and proliferation, and in embryonic development [34].

CHD complexes demonstrate significant diversity. There are nine types of CHD complexes that could be subdivided in three subclasses (CHD1-2, CHD3-4, and CHD5-9), based on the presence of additional functional domains within the ATPase subunit [35]. The best studied member of the CHD family is the nucleosome remodeling and deacetylase (NURD) complex that deacetylates specific genes during development, leading to their transcriptional repression. The NURD complex includes CHD3 or CHD4 subunits, histone deacetylases HDAC1 or HDAC2, lysine-specific histone demethylase 1A (LSD1), and methyl CpG-binding domain (MBD) proteins [36].

Loss-of-function mutations are also common in CHD complexes and have been described in different diseases. Loss of CHD4 expression was found in 56.4% of gastric cancers and in 55.7% of colorectal cancers, whereas *CHD8* was mutated in 35.7% of gastric cancers and in 28.6% of colorectal cancers [1]. *CHD7* is mutated in lung cancers of cigarette smokers [37] and in the CHARGE syndrome, a sporadic autosomal-dominant genetic disorder characterized by a number of birth defects including atrioventricular septal abnormalities [38]. The CHD subunits play essential roles during neurodevelopment [11]. *CHD3* was recently found to be mutated in the Snijders Blok-Campeau syndrome, characterized by developmental delays, impaired speech and language skills, macrocephaly, and characteristic facial features [39]. Mutations in *CHD8* promote the development of the autism spectrum disorders [40].

### 2.4. The INO80/SWR1 Complexes

The INO80/SWR1 family includes three major complexes. Each of them has a unique ATPase unit (INO80, SRCAP or p400) and two ATP-driven helicases, RUVBL1 and RUVBL2 [41]. INO80 can activate transcription and DNA repair [7]. Upon acetylation of the histone H4 and H2A-tails by the p400-TRRAP-TIP60 complex, INO80 and SRCAP complexes replace histone H2A with its homolog H2A.Z, which leads to reduced stability or nucleosome sliding [41]. Nucleosomes with histone H2A.Z are commonly concentrated near the transcription start sites, and the removal of H2A.Z from DNA by the histone chaperone ANP32E and INO80 is a first step in DNA repair [42].

The INO80 is overexpressed in melanoma, cervical cancer, and leukemia [1,37]. In addition, INO80 and another subunit INO80B are upregulated in non-small-cell lung cancer and correlated with prognosis of the disease [1]. Helicases RUVBL1 and RUVBL2 are overexpressed in colon cancer and bladder cancer, respectively [37].

Genetic defects of the components of the INO80 complex, YY1AP1, are associated with the fibromuscular dysplasia, a non-atherosclerotic and non-inflammatory arterial disease that is a part of the Grange Syndrome. The loss of YY1AP1 leads to an increased p21/WAF/CDKN1A levels, and consequently, to the G1 and G2 growth arrest of vascular smooth muscle cells [43].

## 3. NcRNAs as Epigenetic Regulators

NcRNAs are functional transcripts that are not translated into proteins, but instead regulate gene expression at the transcriptional or post-transcriptional level [44]. Protein-coding genes in the human genome comprise approximately 2% of the entire genomic sequence, whereas more than 80% of the genome is pervasively transcribed and produces thousands of ncRNAs [45]. They are localized in transcriptionally active parts of the genome and therefore can be involved in fundamental cellular processes, such as transcription and translation of coding genes, protein synthesis, and many others [46]. Abnormal expression of ncRNAs was described in many pathological conditions, including cancer, cardiovascular and neurodegenerative diseases, rheumatoid arthritis, and diabetes [47].

NcRNAs can be classified according to their function into housekeeping and regulatory ncRNAs. Housekeeping ncRNAs include ribosomal RNAs (rRNAs) and transfer RNAs (tRNAs) that participate in protein synthesis and the group of ncRNAs localized in nucleus, such as small nuclear RNAs (snRNAs) and small nucleolar RNAs (snoRNAs). Regulatory ncRNAs are classified based on their length into small (<200 nucleotides) and long (>200 nucleotides) ncRNAs, with further subdivision into groups depending on their genomic origin and the mechanism of action [48].

### 3.1. Post-Transcriptional Regulation by microRNAs

Short ncRNAs include microRNAs (miRNAs), short interfering RNAs (siRNAs), and Piwi-interacting RNAs (piRNAs) [49]. MiRNAs are the best studied group among them. MiRNAs are evolutionary conserved transcripts of 17–25 nt that regulate gene expression on a post-transcriptional level by cleavage, degradation, or repression of translation of specific target mRNAs [5]. They are expressed in almost all tissues and at all stages of development. Currently, 2654 mature human miRNAs have been described (miRbase, release 22.1, 3 October 2018) [50]. Their mechanism of action is determined by the degree of complementarity between the 3′-untranslated region (UTR) of an mRNA and nucleotides positioned 2–7 from the 5’-end of a miRNA, or the so called “seed sequence”. MiRNAs that are completely complementary with their mRNA targets can induce cleavage and degradation. However, they are very rare, whereas most miRNAs target mRNAs with partial complementary and repress its translation without any effect on a mRNA itself [5].

Biogenesis of miRNAs is a multistep process that occurs both in the nucleus and cytoplasm. Upon transcription of miRNA genes by RNA polymerase II, long-capped RNA molecules of primary miRNAs (pri-miRNAs) are cleaved into 60–100 nucleotide-long hairpin precursors (pre-miRNAs) by a double-stranded RNA-specific ribonuclease III (Drosha). Pre-miRNAs are further transported from the nucleus to the cytoplasm by the nuclear export factor exportin 5, where they are processed by another RNase III enzyme Dicer into 18–25 nucleotide-long miRNA duplexes. The duplex is loaded into the RNA-induced silencing complex (RISC), leading to its unwinding, and commonly, the degradation of one of the two strands. However, in some miRNAs both strands can interact with mRNAs, exerting different functions depending on the site of binding [51]. Finally, the mature single-stranded miRNA associated with RISC is guided to the 3′-UTR of its mRNA targets [52]. Some miRNAs bind not only the 3′-UTRs, but also the 5′-UTRs of mRNA targets, exerting different effects. MiR-10 is the one of them [53]. In addition, miRNAs can interact with the coding sequence of mRNA target, like it was described for the miR-155 [54]. Moreover, miRNAs can regulate mRNA expression by non-canonical way, acting as molecular decoys for RNA-binding proteins without incorporation to the RISC [52].

Because of the relatively short complementary “seed sequences” that are required for interaction with a target, each miRNA can target hundreds of mRNAs, thus regulating multiple genes within one signaling pathway or in different pathways simultaneously [46]. Therefore, miRNAs play a critical role in biological processes, such as cell proliferation, differentiation, and apoptosis, and deregulations of their biogenesis and expression are associated with many pathologies, including cancer, cardiovascular diseases, neurodegenerative, and autoimmune disorders [46,55]. The main causes of miRNA dysregulation in pathogenesis are defects in the miRNA biogenesis machinery, amplifications, deletions, or translocations of the miRNA-encoding genes, abnormal epigenetic modifications, or widespread transcriptional repression [52]. The deregulation of miRNAs is a common feature of all types of cancer. As functions of miRNAs significantly depend on their target genes, their aberrations in pathogenesis could have different effects. Thus, the same miRNA can act both as the tumor suppressor when it is downregulated, or as the oncogene when it is overexpressed in tumors, depending on the tissue or cell type, where it exerts its function, cancer type, or even a stage of the disease [52,56].

### 3.2. Numerous Mechanisms of lncRNA Epigenetic Regulation

LncRNAs are transcripts > 200 nucleotides long with limited or no protein-coding capacity. They are located both in the nucleus or cytoplasm and represent the biggest class of the noncoding RNAs (70–90% of the human genome) [5]. Thus, the NONCODE database has estimated the number of human lncRNA transcripts as 173,112 [57].

LncRNAs are transcribed by the same transcriptional machinery, and sometimes from the same DNA regions, as protein-coding genes and can share with them some other features. Among them are a short open reading frame in some lncRNAs, polyadenylation at the 3’-end, and exons, although shorter and less numerous than in protein-coding genes [5]. There are also distinctive features, including a poor sequence conservation of lncRNAs, lower expression levels, and more specific expression patterns in cells and tissues [58].

Depending on the genomic localization of lncRNA genes and their position relative to protein-coding genes, lncRNAs are further subdivided into groups of intergenic, intronic, antisense, bidirectional, and overlapping lncRNAs [59]. Intronic and overlapping sense lncRNAs can produce circular forms due to the appearance of a covalent linkage at the end of the RNA molecule as a result of non-canonical splicing. Circular RNAs (circRNAs) are highly stable and participate in post-transcriptional regulation, acting as miRNA sponges: they bind miRNAs and thus prevent their interaction with the targets [60].

LncRNAs play an important role in the regulation of transcription, translation, splicing, cell growth and differentiation, apoptosis, cell cycle, dosage compensation, imprinting, pluripotency, and control of chromatin structure and modifications [61]. Their involvement in diverse processes in the cell is determined not only by their numerosity, but also by their localization in different subcellular compartments and ability to form secondary and tertiary structures, which enables them to interact with proteins and chromatin through a variety of mechanisms [5].

Being incorporated into complexes with proteins, lncRNAs can act as scaffolds, guides, decoys, and signals. Molecular scaffolds assemble proteins into complexes, initiate a number of biological processes, and guide direct proteins to the specific sites of the genome. Decoys bind proteins and prevent their interaction with target genes, whereas signals mark specific loci and developmental stages to regulate transcription [59]. LncRNAs can also act separately at the post-transcriptional level by sponging miRNAs, interacting with mRNAs to form double-stranded RNAs, or participating in the biogenesis of small ncRNAs [62].

LncRNAs are aberrantly expressed in various diseases, including cancer, neuropsychiatric disorders, and heart failure. Moreover, they play an important role in their development and progression, and their deregulation leads to alterations in numerous signaling pathways and promotes pathological changes in proliferation, differentiation, and apoptosis [63].

## 4. Mutual Regulation of ncRNAs and Chromatin Remodeling Complexes

The organization and packaging of DNA into chromatin is mediated by many factors. Working together, DNA methylation, histone modifications, and ncRNAs determine the specific structure of chromatin and create stable patterns of gene expression. A number of investigations confirm the mutual regulation between epigenetic regulators of different groups, including ncRNAs and chromatin remodelers, and describe the potential mechanisms of their regulation.

NcRNA regulation commonly appears at the post-transcriptional level. Thus, miRNAs can influence chromatin remodelers by targeting mRNAs of the genes coding their subunits [52]. Computation algorithms and databases predict hundreds of miRNAs that potentially target subunits from SWI/SNF, ISWI, CHD, and INO80 complexes, based on the complementarity of their sequences. Some of them have already been validated as the target pairs (Table 1), and often it was done through a correlation analysis of the expression profiles of specific remodeler subunits with miRNAs identified in different types of cancer [6,7].

MiRNAs not only regulate the individual subunit expression level, but can also interact with complementary sequences in gene promoters, representing a platform for the assembly of protein complexes, thus mediating subunit composition in complexes [52]. MiRNAs, being cytoplasmically processed and incorporated into the Argonaute proteins of RISC complexes, can be further imported into the nucleus by Importin 8. These complexes bind to chromosomal DNA sequences and can either activate or inhibit transcription at the targeted promoter. During activation, the recruitment of chromatin-modifying proteins leads to increased H3K4 methylation, whereas transcriptional gene silencing is determined by increased H3K9/K27 methylation [64]. Similar to the process of 3′ -UTR targeting, gene promoters are targeted depending on the sequence homology between miRNAs and mRNAs. For example, miR-373 can activate E-Cadherin (CDH1) and cold-shock domain-containing protein C2 (CSDC2), which contain miR-373 target sites with at least 80% sequence complementarity in their promoters [65].

In contrast to miRNAs, lncRNAs can regulate chromatin remodeling complexes by variable mechanisms. LncRNAs can directly interact with subunits, acting as guides to anchor the chromatin remodeling complexes or as a decoy to keep chromatin modifiers away from specific sites in the genome. They also can be incorporated into complexes, serve as signals for decoding chromatin modifications, and act as molecular scaffolds to assemble the complex for chromatin remodeling [5,6,7]. LncRNAs can regulate protein complex stability by promoting or inhibiting protein degradation [66]. LncRNAs frequently act as molecular sponges of miRNAs when they have miRNA-binding sites, which are complementary with Ago binding sites in miRNAs. In this case, they can bind specific miRNAs and prevent their interaction with the targets, thus affecting their expression levels and forming lncRNA-miRNAs-mRNA regulatory axes [67,68]. A similar mechanism is common for circRNAs [60,69]. Currently, more than 50 lncRNAs have been described to affect chromatin remodeling complexes, both directly and via lncRNA-miRNAs-mRNA regulatory axes. However, it seems, they are much more numerous [16] (Table 1).

**Table 1:** NcRNAs regulating chromatin remodeling complexes and their role in pathogenesis.

NcRNA	Targeted Subunit	Epigenetic Regulatory Mechanism	Role in Pathogenesis	Status in the Disease	Ref.
SWI/SNF Complex					
CASC15	ARID1A	targets miR-221/ARID1A axis	low expression is correlated with unfavorable prognosis in ovarian cancer	↓	[70]
CDKN2B-AS1	BCL11A	specifically binds BCL11A and inhibit MAP4K1	regulates progression of cerebral infarction	↑	[71]
circ_CSPP1	BRD9	targets miR-486-3p/BRD9 axis	promotes proliferation, invasion, and migration of non-small cell lung cancer cells	↑	[72]
circEPSTI1	BCL11A	targets miR-4753/6809-BCL11A axis	prognostic marker and mediator of triple-negative breast cancer progression	↑	[73]
CPS1-IT1	SMARCA4	competitively binds to SMARCA4 and thus inhibits Cyr61	suppresses melanoma cell metastasis	↓	[74]
DGCR5	ARID1A	interacts with ARID1A to promote p21 expression	correlates with better prognosis and inhibits bladder cancer progression	↓	[75]
DLEU1	SMARCD1	Targets miR-490-3p, altering CDK1, CCND1, and SMARCD1	contributes to ovarian carcinoma tumorigenesis and development	↑	[76]
Evf2	SMARCA4	competing with transcription factor DLX1 for binding to SMARCA4, directly inhibits SMARCA4 ATPase and chromatin remodeling activities	may be involved in neurodevelopmental disorders	↑	[77]
GAS5	ARID1A	targets miR-31-5p/ARID1A axis	inhibits the proliferation and invasion of ovarian clear cell carcinoma	↓	[78]
HIF1A-AS1	SMARCA4	binds to SMARCA4	regulates proliferation and apoptosis of vascular smooth muscle cells in vitro, may contribute to the pathogenesis of thoracic aortic aneurysms	↑	[79]
HOTAIR	SMARCB1, ARID1A	affects expression of transcriptional repressor gene *SNAIL*	involved in kidney cancer progression	↑	[80]
HOTTIP	SMARCE1	targets miR-615-3p/SMARCE1 axis	promotes the metastatic potential of ovarian cancer	↑	[81]
IL-7–AS	SMARCA4	recruits HAT p300 followed by recruitment of SWI/SNF complex to activate inflammatory genes (*CCL2*, *CCL5*, and *IL-6*)	-	-	[82]
LINC00163	ARID1A	recruits ARID1A to the promoter of TCF21 and initiates its expression	suppresses lung cancer development	↓	[83]
LINC00982	DPF3	targets miR-765/DPF3 axis	inhibits the proliferation, migration, and invasion of breast cancer cells	↓	[84]
lincRNA Cox2	SMARCA4	recruits the SWI/SNF complex to inflammatory-response genes	-	-	[85]
lncBRM	SMARCA2	associated with SMARCA2 to initiate the SMARCA4/SMARCA2 switch following SMARCA4-embedded BAF complex triggers activation of YAP1	oncogenic role in hepatocellular carcinoma and liver cancer stem cells, positively correlates with tumor severity	↑	[86]
lncFZD6	SMARCA4	recruits SWI/SNF complex to FZD6 promoter	oncogenic role in liver cancer	↑	[87]
lncTCF7	SMARCA4, SMARCC2, SNF5	recruits the SWI/SNF complex to the *TCF7* promoter	oncogenic role in hepatocellular carcinoma, promotes tumorigenesis of liver cancer stem cells	↑	[88]
MALAT1	SMARCA4	complexes with HDAC9 that represses vascular smooth muscle cell genes	mediates smooth muscle dysfunction in thoracic aortic aneurysm	↑	[89]
MANTIS	SMARCA4	prevents SWI/SNF complex binding at *ICAM-1* promoter region	mediates monocyte adhesion to endothelial cells and thus potentially atherosclerosis development	↓	[90]
MEG3	SMARCB1	targets miR-6088/SMARCB1 axis	acts as tumor-suppressor in glioma cells	↓	[91]
miR-139-5p	SMARCA4	directly targets SMARCA4	involved in pathogenesis of asthma	↓	[92]
miR-140-3p	BRD9	directly targets BRD9	tumor suppressor in squamous cell lung cancer	↓	[93]
miR-144-3p	SMARCA4, ARID1A	promotes BRG1/NRF2/ARE signaling; directly targets *ARID1A*	involved in cerebral ischemia/reperfusion-induced neuronal injury; promotes cell proliferation, metastasis, sunitinib resistance in clear cell renal cell carcinoma	↑	[94,95]
miR-146a	BCL11A	directly targets *BCL11A*	induces apoptosis in neuroblastoma cells	↓	[96]
miR-155	SMARCA4	directly targets *SMARCA4*, activating STAT3/VEGFC signaling	oncogenic role in natural killer/T-cell lymphoma	↑	[97]
miR-199a	SMARCA2	targets *SMARCA2* to generate a double-negative feedback loop	deregulated (either upregulated or downregulated) in a variety of tumors	↑↓	[98]
miR-199a-5p	SMARCA4	targets *SMARCA4* to activate NRF2/HO-1 signaling	involved in cerebral ischemic/reperfusion injury	↓	[99]
miR-202-5p	SMARCC1	directly targets *SMARCC1*	tumor suppressor in colorectal carcinoma	↓	[100]
miR-21	SMARCA4	directly targets *SMARCA4*	significantly elevated in the majority of human tumors, regulates proliferation, survival, and migration	↑	[101]
miR-221/222	ARID1A	simultaneously targets *ARID1A*	enhance proliferation and invasion of cervical cancer cells	↑	[102]
miR-223	SMARCD1	directly targets *SMARCD1*	involved in atypical proliferative serous tumor and high-grade ovarian serous carcinoma	↑	[103]
miR-223-3p	ARID1A	directly targets *ARID1A*	promotes cell proliferation and invasion; involved in *Helicobacter pylori* CagA-induced gastric carcinogenesis and progression	↑	[104,105]
miR-296-5p	SMARCA4	regulates BRG1/SALL4 axis	suppresses stem cell potency of hepatocellular carcinoma cells	↓	[106]
miR-29a	SMARCE1	directly targets *SMARCE1*	induces hepatitis B virus replication in hepatocellular carcinoma	↑	[107]
miR-31	ARID1A	directly targets *ARID1A*	enhances the oncogenicity and stemness of head and neck squamous cell carcinoma; prognostic factor and oncomir in cervical cancer	↑	[108]
miR-320c	SMARCC1	directly targets *SMARCC1*	regulates gemcitabine-resistance in pancreatic cancer	↑	[109]
miR-372	SMARCC1	targets *SMARCC1*, *MECP2*, *CDKN1*, *RBL2*, *RHOC*, and *TGFBR2*, increasing primordial germ cell-like cell production	-	-	[110]
miR-486-3p	BCL11A	directly targets *BCL11A*	might contribute to the β-thalassemia	↑	[111]
miR-490-3p	SMARCD1	directly targets *SMARCD1*	significantly downregulated in human gastric cancer	↓	[112]
miR-501-3p	BCL7A	directly targets *BCL7A*	promotes osteosarcoma cell proliferation, migration, and invasion	↑	[113]
miR-574-5p	BCL11A	targets *BCL11A* and *SOX2* to inhibit the SKIL/TAZ/CTGF axis	attenuates proliferation, migration, and EMT in triple-negative breast cancer	↓	[114]
miR-6511b-5p	SMARCA4	directly targets *SMARCA4* and thus promotes methylation of *CD44*	suppresses metastasis of proficient mismatch repair (pMMR) colorectal cancer	↓	[115]
miR-802	SMARCE1	directly targets *SMARCE1*	induces hepatitis B virus replication in hepatocellular carcinoma	↑	[116]
miR-9* and miR-124	ACTL6A	directly targets *ACTL6A*	selectively expressed in post-mitotic neurons; being mutated, leading to defective activity-dependent dendritic outgrowth in neurons	↓	[117]
miR-99a-5p	SMARCD1	directly targets *SMARCD1*	induces cellular senescence in gemcitabine-resistant bladder cancer	↓	[118]
MVIH	ARID1A	directly targets ARID1A and affects *CDKN1A* transcription	represses hepatocellular carcinoma cell proliferation and migration	↓	[119]
Myheart	SMARCA4	binds to SMARCA4 preventing DNA binding of SWI/SNF complex	protects the heart from pathological hypertrophy	↓	[120]
NEAT1	SMARCA2, SMARCA4	directly targets either SMARCA2 or SMARCA4, facilitates the organization of paraspeckle proteins	regulates sensitivity of cancer cells to chemotherapy and p53 reactivation therapy	↑	[121]
SBF2-AS1	SMARCD1	targets miR-520f-3p/SMARCD1 axis	promotes abdominal aortic aneurysm formation	↑	[122]
SChLAP1	SMARCA4, SNF5	associates with SMARCA4, preventing SWI/SNF complex binding; directly binds to hSNF5 and impairs proper SWI/SNF regulation of gene expression	promotes aggressive prostate cancer; leads to tumor cell invasion and metastasis in prostate cancers	↑	[123,124]
SWINGN	SMARCB1	transcriptional activation of several genes (e.g., *GAS6*, *PDGFRB*, and *COL1A1*)	oncogenic in lung squamous carcinomas and breast invasive carcinoma	↑	[125]
THRIL	SMARCA4	targets miR-99a/SMACA4 axis	promotes hypoxia-induced cell injuries	↑	[126]
TUG1	SMARCA4	directly targets SMARCA4 and inhibits degradation	mediates the protective effect of propofol against hepatic ischemia/reperfusion injury	↑	[127]
uc.291*	ACTL6A	binds with ACTL6A and disturbs BAF-ACTL6A binding	-	-	[128]
uc.57*	BCL11A	binds to BCL11A and inhibits PI3K/AKT and MAPK signaling pathways	promotes tamoxifen resistance in breast cancer	↓	[129]
UCA1	SMARCA4	binds SMARCA4 and impairs both binding of SMARCA4 to the promoter region of p21 and chromatin remodeling activity of SMARCA4	promotes bladder cancer cell proliferation	↑	[130]
XIST	SWI/SNF	directly binds to BRG1 and expels BRG1 from the Xi-genes regions	-	-	[131]
ISWI					
circ_0006168	RBBP7	targets miR-384/RBBP7 axis	contributes to cell proliferation, migration, and invasion in esophageal cancer	↑	[132]
circ_0102231	RBBP4	targets miR-145/RBBP4 axis	promotes non-small cell lung cancer cell proliferation	↑	[133]
circ-DONSON	SMARCA1	recruits the SMARCA1-NURF complex to the *SOX4* promoter, leading to enrichment of the active markers H3K27ac and H3K4me3 on the promoter and activation of *SOX4* transcription	facilitates tumor cell development in gastric tumor cells	↑	[134]
DLEU1	SMARCA1	recruits the SMARCA1-NURF complex to activate *KPNA3*	contributes to colorectal cancer progression	↑	[135]
FGD5-AS1	CDCA7	targets miR-302e/CDCA7 axis	promotes colorectal cancer cell proliferation, migration, and invasion	↑	[136]
LINC00885	BAZ2A	targets miR-3150b-3p/BAZ2A axis	promotes cervical cancer progression	↑	[137]
lnc pRNA	BAZ2A	establishes heterochromatin at ribosomal RNA genes e.g., RNA45SN2 (45S pre-rRNA)	-	-	[138]
lncKdm2b	SATB1-ISWI	promotes recruitment of NURF complex to Zfp292 via recruitment of chromatin organizer SATB1	-	-	[139,140]
lncRNA HOXA-AS2	RBBP4	targets miR-885-5p/RBBP4 axis	promotes glioblastoma carcinogenesis	↑	[141]
miR-146b-5p	SMARCA5	directly targets *SMARCA5*	suppresses the malignancy of glioma cells	↓	[142]
miR-151-5p	SMARCA5	directly targets *SMARCA5*	overexpressed in PARP1-upregulating BRCA-germline mutated and sporadic breast tumors	↑	[143]
miR-429	RBBP4	targets the RBBP4/E2F1/OCT4 axis	promotes hepatocarcinogenesis	↑	[144]
NEXN-AS1	BAZ1A	targets BAZ1A upregulates *NEXN* expression	associated with atherosclerosis-related diseases	↑	
NMR	BPTF	NURF complex recruitment promoting ERK1/2 signaling pathway	promotes tumor progression in esophageal squamous cell carcinoma	↑	[145]
Paupar	TRIM28	forms Paupar-KAP1-PAX6 complex, promoting KAP1 (H3K9me3 deposition) recruitment at a subset of distal targets	-	-	[146]
CHD					
ANRIL	HDAC1	targets miR-34a/HDAC1 axis	promotes cell proliferation, migration, and invasion during acute myeloid leukemia	↑	[147]
ARAP1-AS1	HDAC2	targets miR-2110/HDAC2/PLIN1 axis	accelerates cell proliferation and migration in breast cancer	↑	[148]
CHASERR	CHD2	transcriptional interference between CHASERR and CHD2 results in negative feedback loop	may be involved in neurological disease	↑	[149]
circ_0006528	CHD4	targets miR-1236-3p/CHD4 axis	promotes cell proliferation, migration, invasion, and adriamycin chemoresistance in breast cancer	↑	[150]
circ_0007396	CHD4	targets miR-767-3p-CHD4 axis	involved in the progression and carcinogenesis of gastric cancer	↑	[151]
circ_0039411	MTA1	targets miR-1205/MTA1 axis	promotes tumorigenesis and progression of papillary thyroid cancer	↑	[152]
circ-NCOR2	MTA2	targets miR-516a-5p/MTA2 axis	accelerates papillary thyroid cancer progression	↑	[153]
circPDZD8	CHD9	targets miR-197-5p/CHD4 axis	promotes gastric cancer progression	↑	[154]
circ-UBAP2	CHD2, MTA1	targets miR-144/CHD2 axis; targets miR-661/MTA1 axis	promotes the progression of ovarian cancer; predicts poor prognosis and promotes triple-negative breast cancer progression	↑	[155,156]
circ-SFMBT2	CHD1	targets miR-30d-5p/CHD1 axis	oncogenic role in colorectal adenocarcinoma	↑	[157]
ELFN1-AS1	MTA1	targets miR-1250/MTA1 axis	promotes cell proliferation, migration, and invasion, and induce apoptosis in colorectal cancer	↑	[158]
HOTAIR	MTA2	targets miR-326/MTA2 axis	promotes the invasion and metastasis of oral squamous cell carcinoma	↑	[159]
let-7	CHD7	directly targets *CHD7*	-	-	[160]
LINC00963	MTA1	targets miR-766-3p/MTA1 axis	oncogene in bladder cancer	↑	[161]
LINC01410	CHD7	targets miR-23c/CHD7 axis	promotes endometrial cancer progression	↑	[162]
LINC02535	CHD1	targets miR-30d-5p/CHD1 axis	induces colorectal adenocarcinoma progression	↑	[163]
MATN1-AS1	CHD1	targets miR-200b/c/429/CHD1 axis	promotes glioma progression	↑	[164]
miR-1236-3p	MTA2	directly targets *MTA2*	inhibits invasion and metastasis in gastric cancer	↓	[165]
miR-130b-3p	CHD9	directly targets *CHD9*	promotes colorectal cancer progression	↑	[166]
miR-141-3p	CHD8	interacts with the *CHD8*	contributes to the regulation of cardiomyocyte apoptosis and consequent cardiovascular diseases	↑	[167]
miR-148b	MTA2	directly targets *MTA2*	suppresses proliferation, migration, and invasion of nasopharyngeal carcinoma cells	↓	[168]
miR-208a	CHD9	targets *CHD9* through Notch/NF-kappa B signal pathways	involved in development of cardiovascular disease	↑	[169]
miR-22-3p	CHD9	directly targets *CHD9*	involved in development of hypertension	↑	[170]
miR-30	MTA1	directly targets *MTA1*	inhibits the progression of osteosarcoma	↓	[171]
miR-30a-5p	CHD1	directly targets *CHD1*	enhances cisplatin sensitivity of ovarian cancer cells through the Wnt/β-catenin pathway	↓	[172]
miR-30c-5p	MTA1	directly targets *MTA1*	suppresses migration, invasion, and epithelial to mesenchymal transition of gastric cancer	↓	[173]
miR-421	MTA1	directly targets *MTA1*	inhibits proliferation and metastasis of colorectal cancer	↓	[174]
miR-548b	MTA2	directly targets *MTA2*	inhibits the proliferation and invasion of malignant gliomas	↓	[175]
miR-589	MTA2	directly targets *MTA2*	tumor suppressor in breast cancer	↓	[176]
PAPAS	CHD4	recruits the NuRD complex to rRNA genes through an interaction with *CHD4* upon hypotonic stress induction	-	-	[177]
SNHG15	HDAC2	targets miR-490-3p/HDAC2 axis	accelerates the development of hepatocellular carcinoma	↑	[178]
TTC39A-AS1	MTA2	targets miR-483-3p/MTA2 axis	promotes breast cancer	↑	[179]
INO80					
ANRIL	YY1	recruits YY1 to promoter loci of IL6 and IL8	involved in coronary artery disease	↑	[180]
CASC7	ING3	targets miR-21/ING3 axis	inhibits the proliferation and migration of colon cancer cells	↓	[181]
circMYO10	RUVBL1	targets miR-370-3p/RUVBL1 axis to enhance the transcriptional activity of β-catenin/LEF1 complex	promotes osteosarcoma progression	↑	[182]
CR933609	INO80D	targets miRNA-5096/INO80D axis	tumor-suppressor in non-small cell lung cancer	↓	[183]
DRAIC	UCHL5	promotes NFRKB (INO80G) ubiquitination-mediated degradation	inhibits proliferation and metastasis of gastric cancer cells	↓	[184]
HAND2-AS1	INO80	recruits the INO80 complex to the *BMP-R1A* and *Nkx1-2* promoter	promotes liver cancer stem cell self-renewal	↑	[185]
HOTAIR	YY1	sponges miR-1/miR-206 and targeting YY1	promotes medulloblastoma growth, migration, and invasion	↑	[186]
LCTS5	INO80	binds to INO80, inhibiting binding to enhancer regions near lung cancer associated genes	inhibits non-small cell lung cancer	↓	[187]
LINC00668	YY1	targets miR-532-5p/YY1 axis	promotes cell proliferation, migration, invasion ability and EMT process in hepatocellular carcinoma	↑	[188]
LINC00839	RUVBL1	recruits RUVBL1/Tip60 complexes to activate NRF1	promotes colorectal cancer progression	↑	[189]
LINC00899	YY1	targets miR-744-3p/YY1 axis	promotes progression of acute myeloid leukemia	↑	[190]
linc01134	YY1	targets miR-324-5p/IGF2BP1/YY1 axis	regulates hepatocellular carcinoma progression	↑	[191]
linc-MYH	INO80	acts as a selective molecular switch in trans and configures INO80	-	-	[192]
linc-YY1	YY1	evicts YY1/Polycomb repressive complex (PRC2) from target promoters	-	-	[193]
lncAKHE	YEATS4	activates NOTCH2 signaling	enhances cell growth and migration in hepatocellular carcinoma	↑	[194]
lncKdm2b	SRCAP	recruits SRCAP complex to Zbtb3 gene, activating expression of this transcription factor	-	-	[139]
miR-129*	MCRS1	targets MCRS1/miR-155 axis	promotes the epithelial-mesenchymal transition and metastasis in non-small cell lung cancer	↑	[195]
RNCR3	BRD8	targets miR-185-5p/BRD8 axis	promotes prostate cancer progression	↑	[196]
SPAG5-AS1	YY1	targets miR-769-5p/YY1 axis	involved in diabetic nephropathy		[197]
TUG1	miR-145, YY1	interfers PRC2 complex	promotes glioma	↑	[198]

↑—the ncRNA is upregulated in the mentioned pathological condition; ↓—the ncRNA is downregulated in the mentioned pathological condition.

Alternatively, ncRNAs are also regulated by chromatin remodeling complexes, despite fewer mechanisms of such regulation having been described. Nevertheless, some chromatin remodelers from main subfamilies play an important role in the pre-transcriptional regulation of ncRNAs [199].

### 4.1. NcRNAs and SWI/SNF Complexes: The Most Investigated Mutual Regulation

Most of the already described interactions between ncRNAs and chromatin remodelers refer to SWI/SNF complexes. However, this probably appears not because of less common interactions between ncRNAs and other chromatin remodeling complexes, but because of a bigger number of studies devoted to SWI/SNF complexes [199].

**Figure 1:**
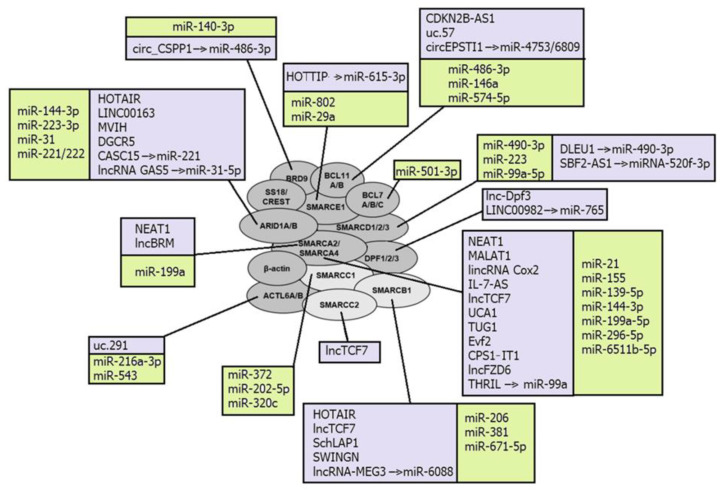
Regulation of BAF complexes of the SWI/SNF family by ncRNAs. The scheme demonstrates miRNAs (highlighted in green) and lncRNAs, including circRNAs (highlighted in lilac) that affect specific subunits of the chromatin remodeling complex. All miRNAs target respective mRNAs directly; for lncRNAs both the direct and indirect effect via lncRNA-miRNA-mRNA regulatory axis was presented.

A number of lncRNAs were confirmed as regulating the catalytic subunits of SWI/SNF complexes, SMARCA4 and SMARCA2 (Figure 1). LncRNA urothelial carcinoma associated 1 (UCA1) directly binds SMARCA4 and impairs both the binding of SMARCA4 to the promoter region of *p21* and chromatin remodeling activity of SMARCA4. Being overexpressed, UCA1 promotes bladder cancer cell proliferation [130]. Another lncRNA HIF1A-AS1 also binds to SMARCA4, and thus regulates cell proliferation and apoptosis in tumors [79]. LncBRM demonstrates an aberrantly high expression level in liver cancer stem cells and hepatocellular carcinoma, where it positively correlates with tumor severity. LncBRM was found to be associated with SMARCA2 to initiate the SMARCA4/SMARCA2 switch, following the activation of YAP1 signaling by the SMARCA4-embedded BAF complex [86].

Nuclear paraspeckle assembly transcript 1 (NEAT1) directly interacts with either SMARCA4 or SMARCA2 in the process of the lncRNA-dependent nuclear body assembly, and forms the paraspeckle structures that modulate the replication stress response and chemosensitivity. Silencing of NEAT1 expression prevents paraspeckle formation, sensitizing preneoplastic cells to DNA-damage-induced cell death and impairing skin tumorigenesis [121]. NEAT1 has been described as aberrantly expressed in breast cancer, gastric cancer, lung cancer, and leukemia [200,201,202,203].

Other SWI/SNF subunits could also be targeted by ncRNAs. For example, lncRNA uc.291 binds with ACTL6A and disturbs BAF-ACTL6A binding, thus affecting the expression of several genes in skin keratinocytes [128]. ARID1A binding with the lncRNAs LINC00163 and DGCR5 stimulates transcription of genes *TCF21* and *p21*, respectively [75,83], whereas SMARCB1 interacts with lncRNAs HOTAIR and SWINGN [80,125].

The second chromosome locus associated with prostate 1 (SChLAP1) is one of the lncRNAs that can be incorporated into SWI/SNF complexes, acting as a scaffold to assemble the complex for chromatin remodeling. SChLAP1 directly binds to hSNF5 and impairs proper SWI/SNF regulation of gene expression, which leads to tumor cell invasion and metastasis in prostate cancers [124].

LncTCF7 recruits several subunits of SWI/SNF complexes, namely SMARCA4, SMARCB1, and SMARCC2, to the *TCF7* promoter. Such interaction is enabled by a region of 200 nts at the 3′-UTR of lncTCF7, which is necessary to bind these three subunits, and is a stable stem-loop secondary structure. LncTCF7 is overexpressed in hepatocellular carcinoma, where it promotes the tumorigenesis of liver cancer stem cells [88].

LncRNAs can also recruit inflammatory transcription factors into SWI/SNF complexes. Thus, lincRNA Cox2 is incorporated into SWI/SNF complexes in cells after bacterial lipopolysaccharide stimulation, and the lincRNA Cox2-SWI/SNF complex further modulates the NF-κB subunits assembling into SWI/SNF complexes and promotes the transcription of late inflammatory genes in macrophages [85].

Among the miRNAs directly targeting and post-transcriptionally regulating gene-coding subunits of SWI/SNF complexes are: miR-155 that inhibits *SMARCA4* expression, thus activating downstream STAT3/VEGFC signaling and promoting lymphangiogenesis [97]; miR-490-3p that targets the 3′-UTR of *SMARCD1* and, being aberrantly overexpressed, represses BAF tumor suppressor activity in gastric cancer [112]; and miR-144-3p that promotes cell proliferation, metastasis, and sunitinib resistance in clear cell renal cell carcinoma by downregulating *ARID1A* [94]. MiRNAs miRNA9* and miRNA124 are involved in the exchange of subunits and alteration of subunit composition in the BAF complex during mammalian neural development. During differentiation, they mediate the downregulation of ACTL6A from the neural progenitor BAF complex to enable its swapping with ACTL6B, referred to as neural BAF. Before differentiation, these miRNAs are repressed by the transcriptional repressor REST, which is downregulated by the unliganded retinoic acid receptor (RAR) complex at the onset of differentiation [117].

Some lncRNAs have been described as regulators of SWI/SNF complexes by sponging miRNAs that directly target its subunits. LncRNA MEG3 sponges miR-6088, targeting *SMARCB1*, and thus acts as the tumor-suppressor in glioma cells [91], whereas lncRNA GAS5 inhibited the cell viability and invasion of ovarian clear cell carcinoma through the activation of *ARID1A* by sponging miR-31-5p [78]. CircRNAs circ_CSPP1 acts by a similar mechanism and regulates the development of non-small cell lung cancer through the miR-486-3p/BRD9 axis [72].

Because of the ability to mobilize or eject nucleosomes to regulate chromatin accessibility at the regions where they were recruited, SWI/SNF complexes can both promote and repress non-coding transcription, associated with regulatory elements [199]. For example, the embryonic stem cell-specific BAF (esBAF) complex is localized in gene regulatory elements within embryonic stem cells and reinforces the occupancy of the enhancer to suppress eRNA, or promoter flanking nucleosomes to suppress PROMPT expression [204]. Therefore, SWI/SNF complexes can regulate the expression of both protein-coding genes and ncRNAs. For example, it was confirmed that SMARCA4 regulates miRs-143/145 and miR-133 in smooth muscle. SMARCA4 is required for myocardin to induce the binding of serum response factor (SRF) to the regulatory region of miR-143/145, which is sufficient to activate its transcription. In contrast, the regulation of miR-133 expression by SMARCA4-containing chromatin remodeling complexes is partially SRF-dependent and requires other factors, cooperating with SRF to activate transcription [205]. The SMARCA4 also regulates miR-550a-5p, thus starting the miR550a-5p/RNF43/Wnt signaling axis. SMARCA4 acts as a tumor suppressor and, being downregulated, promotes colorectal cancer metastasis via this axis [206]. Another catalytic subunit of SWI/SNF complexes SMARCA2 positively regulates the transcription of miR-302a-3p, which acts as a metastasis-promoting miRNA in pancreatic cancer cells. As miR-302a-3p directly targets *SOCS5* to boost STAT3 phosphorylation and induce the transcription of *STAT3* target genes, SMARCA2 starts the miR-302a-3p/SOCS5/STAT3 signaling axis and thus potentiates pancreatic cancer metastasis [207].

### 4.2. NcRNAs and ISWI Complexes: Guides, Scaffolds and Sponges

**Figure 2:**
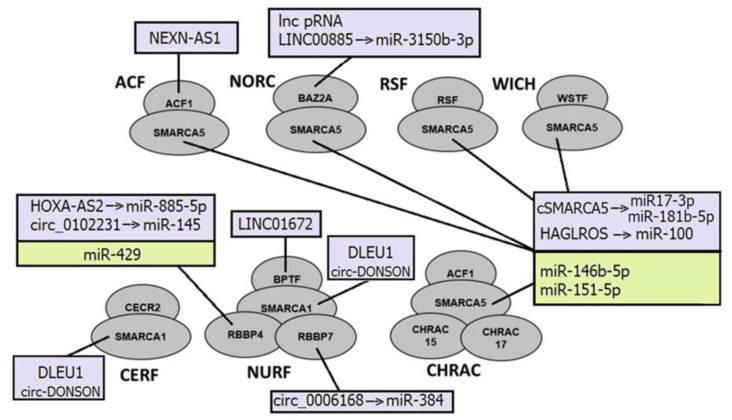
Regulation of ISWI complexes by ncRNAs. The scheme demonstrates miRNAs (highlighted in green) and lncRNAs, including circRNAs (highlighted in lilac) that affect specific subunits of the chromatin remodeling complex. All miRNAs target respective mRNAs directly; for lncRNAs both the direct and indirect effect via lncRNA-miRNA-mRNA regulatory axis was presented.

NcRNAs can directly bind to subunits of ISWI complexes and serve as guides to anchor ISWI complexes. For example, lncRNA NEXN-AS1 recruits BAZ1A (ACF complex) to NEXN, thus upregulating its expression [208] (Figure 2).

They also can be incorporated into ISWI complexes and function as scaffolds to assemble the complex for chromatin remodeling. Circ-DONSON directly interacts with SMARCA1 and recruits the SMARCA1-NURF complex to the *SOX4* promoter. That leads to the enrichment of the active markers H3K27ac and H3K4me3 on the promoter, and the activation of *SOX4* transcription, thus facilitating tumor cell development in gastric tumor cells [134]. This SMARCA1-NURF complex is also recruited by another lncRNA, lnc-DLEU1, to the *KPNA3* promoter in colorectal cancer, where it initiates *KPNA3* expression via H3K27ac enrichment and promotes tumor cell proliferation and migration [135]. The cSMARCA5, a circRNA derived from exons 15 and 16 of the *SMARCA5* gene, acts as a sponge for miR-17-3p and miR-181b-5p to upregulate TIMP3. cSMARCA5 is upregulated in prostate cancer [209]. MiR-146b-5p and miR-151-5p also target *SMARCA5* and thus contribute to the malignant progression of gliomas and breast cancer, respectively [142,143].

The regulation of ncRNAs by ISWI complexes is not well investigated. However, it was described that they modulate nucleosome positioning to establish evenly spaced arrays of nucleosomes, suppressing intergenic and intragenic ncRNA expression [199].

### 4.3. NcRNAs and CHD Complexes: CHD Subunits and Potential of Reverse Regulation

**Figure 3:**
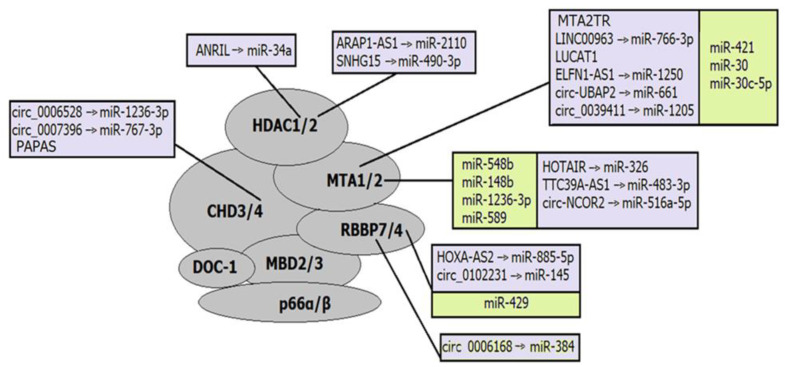
Regulation of CHD complexes by ncRNAs. The scheme demonstrates miRNAs (highlighted in green) and lncRNAs, including circRNAs (highlighted in lilac) that affect specific subunits of the chromatin remodeling complex. All miRNAs target respective mRNAs directly; for lncRNAs both the direct and indirect effect via lncRNA-miRNA-mRNA regulatory axis was presented.

Not so many ncRNAs that interact with the CHD family complex have been described. Some of them regulate CHD subunits (Figure 3). For example, lncRNA PAPAS recruits the NuRD complex to rRNA genes through the interaction with CHD4 upon hypotonic stress induction [177]. LncRNA CHASERR affects the CHD2 complex by a negative regulation loop. The *CHASERR* gene and CHD2 are located on the same strand at chr15q26; therefore, the CHASERR transcript can be bound by the CHD2 protein and regulates the transcription of the *CHD2* gene [149].

The CHD subunits of almost all types were validated as direct targets of miRNAs. For example, miR-30a-5p targets *CHD1* and enhances the cisplatin sensitivity of ovarian cancer cells through the Wnt/β-catenin pathway [172]; miR-141-3p contributes to the regulation of cardiomyocyte apoptosis and consequently cardiovascular diseases through interaction with *CHD8* [167], whereas miR-130b-3p promotes colorectal cancer progression by targeting *CHD9* [166]. Some of these miRNAs are involved in epigenetic regulatory axes, where they, in turn, are regulated by lncRNAs or circRNAs. Some of such regulatory axes have been described in pathological conditions. Thus, lncRNA LINC02535 was confirmed to induce colorectal adenocarcinoma progression via targeting miR-30d-5p/CHD1 axis [163], and circ-SFMBT2 sponges miR-30d-5p that directly targets CHD1 and therefore plays an oncogenic role in colorectal adenocarcinoma [157].

CHD complexes demonstrate significant potential as ncRNA regulators. Thus, CHD1 arranges nucleosomes into evenly spaced arrays in the wake of the transcriptional machinery, suppressing expression of intragenic cryptic ncRNAs [199,210], whereas CHD4 regulates rRNA expression through modulating nucleosome positioning [211]. In addition, CHD7 and CHD8 are localized to enhancer elements in human cell lines. Although the functions of these complexes at enhancers remain unknown, the localization of them to each target location is critical for the transcriptional activation of nearby genes [199,212,213].

### 4.4. NcRNAs and INO80 Complexes: Mediating Histone Modifications

**Figure 4:**
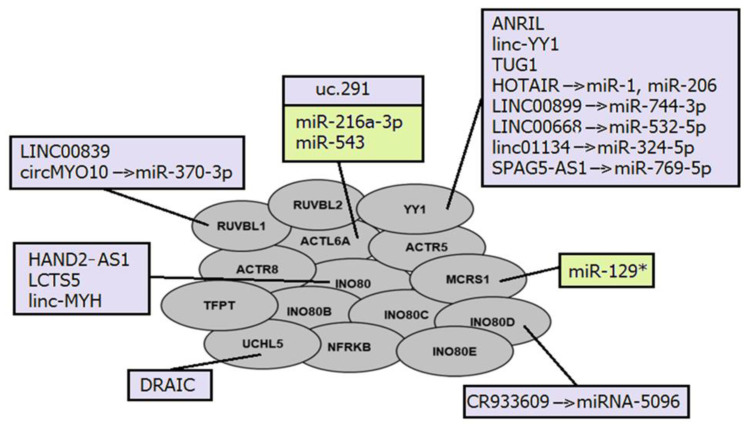
Regulation of INO80 complexes by ncRNAs. The scheme demonstrates miRNAs (highlighted in green) and lncRNAs, including circRNAs (highlighted in lilac) that affect specific subunits of the chromatin remodeling complex. All miRNAs target respective mRNAs directly; for lncRNAs both the direct and indirect effect via lncRNA-miRNA-mRNA regulatory axis was presented.

The interactions between ncRNAs and INO80 complexes are generally similar to those already mentioned for other nucleosome remodeling complexes. Several lncRNAs have been described as regulators of INO80 subunit expression (Figure 4). LncRNA UCHL5 mediates the de-ubiquitination of NFRKB (INO80G), which is prevented by its interaction with another lncRNA DRAIC [184]. In addition, lncRNA PTCSC3 inhibits INO80 expression by negatively regulating STAT3 [214].

LncRNA LCTS5 and HAND2-AS1 both interact with the INO80 subunit; however, they have opposite effects. Thus, LCTS5 inhibits INO80 complex recruitment, whereas HAND2-AS1 stimulates it [185,187,215]. Two another lncRNAs ANRIL and linc-YY1 also interact with the transcription factor YY1 in a similar manner, resulting in the recruitment of IL6/IL8 to promoter loci or eviction of the Polycomb repressive complex 2 (PRC2) regulated promoters, respectively [180,193].

The cytoplasmic lncRNA CR993309 was found to be complementary to the 3′-UTR of the *INO80D* gene with 99% sequence homology to an approximately 9 kb region. It is involved in the post-transcriptional regulation of the INO80D subunit in epithelial cells by sponging miRNA-5096 [183].

Members of the INO80 family regulate PROMPT expression through nucleosome editing, but suppress heterochromatin-associated transcripts through less clearly understood mechanisms than SWI/SNF complexes. It was suggested that INO80 complexes have a conserved role in ncRNA suppression in the wide genome, influencing several ncRNA species, which may be related to the variant of canonical histone H2A, H2AZ [199].

## 5. NcRNAs and Chromatin Remodeling Complexes as Diagnostic Markers and Therapeutic Targets

The association of chromatin remodelers with diseases was first found in 1999, when the subunit SNF5 (SMARCB1) of the SWI/SNF remodeling complex was discovered to be inactivated in rhabdoid tumors [216]. This finding led to the consequent assumption that other chromatin remodeling complexes and their subunits could also be involved in development and progression of various diseases [9]. Deregulation of ncRNAs resulting in aberrant gene expression and therefore involved in pathological conditions was also confirmed by numerous investigations.

An important role in virtually all pathological processes, the early appearance of aberrations and their alterations in different stages of a pathogenesis, make epigenetic regulators prominent diagnostic and prognostic biomarkers, whereas their flexibility and reversibility make them an attractive target for therapy [217].

As genes, coding subunits of chromatin remodeling complexes are often mutated, differentially expressed in diseases, and associated with response to treatment, it is not unexpected that some of their mutations may have diagnostic and prognostic value [1]. NcRNAs also offer some advantages as biomarkers. Specifically, they are more tissue-specific than protein-coding genes and stable at high temperatures, strong acidic or basic conditions, and long-term storage, and therefore could be detected in a wide range of samples, including body fluids and formalin-fixed paraffin-embedded samples. In addition, some of them could be predictive factors for the clinical response to therapies [5]. However, application of ncRNAs in clinical practice has limitations because of their low expression levels, variability of their expression in different conditions (which can result in biases even when a panel of several transcripts is used), and significant regional differences in ncRNA profiling [5].

A recent investigation revealed that inhibition of specific chromatin remodeling complexes could improve treatment efficiency [17]. Therefore, the chemical and pharmacological agents that can directly block complexes and their subunits are being actively investigated. The development of effective chemical drugs, directly targeting subunits of chromatin remodeling complexes to enhance a synthetic lethality, depends on the presence of certain regions in the protein molecule that can be used for interaction with chemical compounds. Therefore, the development of targeted drugs is mainly focused on subunits with ATPase domains and bromodomains. The subunits SMARCA2/4, BRD7, BRD9, and PBRM1 of the SWI/SNF complex contain bromodomains, which make them potential targets for direct inactivation of the complex. Bromodomains are highly conserved protein–protein interaction modules that recognize acetylated lysines on histone tails, contributing to the expression of target genes. Interaction with acetyl-lysines occurs through a specific pocket that can be used to bind to a chemical inhibitor [218]. The bromodomains of the BRD7/9, SMARCA2/4, and PB1 read the acetylation marks H3K14ac, H3K27ac, or H3K9ac, thereby recruiting SWI/SNF complexes to target gene regions, activating their expression [14]. Despite being optimal targets for inactivation of specific SWI/SNF subunits, bromodomains have been identified in many human proteins and are not always associated with chromatin remodeling. Bromodomain inhibitors have been proposed for the treatment of tumors, and some of them are already at the I-II stages of clinical trials. Their use as specific inhibitors of the SWI/SNF chromatin remodeling complex has not been widely used, however, since specific bromodomain inhibitors have not demonstrated the ability to induce synthetic lethality [219].

The members of the ISWI complex also contain bromodomains and therefore are attractive targets for drug design. Several potent inhibitors were already developed for the bromodomains of ISWI complexes. NVS-CECR2–1 selectively inhibits chromatin binding of the CECR2 bromodomain and displaces CECR2 from chromatin within cells. It exhibits cytotoxic activity against several cancer types, mainly through inducing cell apoptosis. GSK2801 is a selective and cell-active acetyl-lysine competitive inhibitor of BAZ2A/B bromodomains. Although GSK2801 has little effect on growth arrest as a single agent, it shows a strong synergistic effect on triple-negative breast cancer in combination with the BET bromodomain inhibitor (BETi) JQ1. Arylurea (AU1) was the first small molecule selective inhibitor of the BPTF bromodomain. It is selective for BPTF over BRD4, with moderate potency in an in vitro assay. AU1 treatment alters chromatin accessibility, decreases target gene c-MYC chromatin occupancy, weakens proliferative capacity, and leads to G1 arrest in mouse breast cancer cells [26].

Recently, the AU-15330 destructor (PROTAC) has been developed, which cleaves the ATPase subunits of SWI/SNF SMARCA2 and SMARCA4 [220]. It is a highly specific and VHL-dependent inhibitor of SWI/SNF ATPase components and exhibits cytotoxicity in tumors at low concentrations. It has been demonstrated that complete inactivation of SWI/SNF ATPases induces a targeted and rapid loss of chromatin availability to *AR*, *FOXA1*, *MYC*, and *ERG*, weakening their transcription, as well as related genes, suppressing the enhancer-related overexpression of driver oncogenes. These results confirm that constant activity of the SWI/SNF remodeling complex is necessary to preserve enhancers in an open nucleosome-free conformation. The treatment was with the SMARCA2/4 destructor-induced significant inhibition of tumor growth in xenograft models obtained from a cell line, multiple myeloma, and prostate cancer. Moreover, no serious toxicity was observed in mice even after prolonged treatment with the SMARCA2/4 destructor [221,222].

In addition to chemical and pharmacological agents that can directly block chromatin remodeling complexes and their subunits, indirect approaches are being actively investigated. One of the strategies for influencing epigenetic regulators associated with chromatin is the additional blocking of proteins of the remodeling complex in tumor tissue to cause the synthetic lethality of tumor cells. Inhibition of the growth of rhabdoid tumors with lack of the SMARCB1, through knockdown of the SWI/SNF catalytic subunit SMARCA4, suggests that the survival of such tumor cells may depend on the residual activity of the SWI/SNF complex. This is confirmed by the existence of mutually exclusive subunits in SWI/SNF complexes [223]. As the ARID1A and ARID1B proteins are mutually exclusive in the SWI/SNF complex, it could be proposed that the survival of tumor cells with *ARID1A* mutations may depend on the presence of *ARID1B* in the residual SWI/SNF complex.

The same mechanism could be applied for SMARCA2 and SMARCA4, since they form mutually exclusive complexes. The survival of SMARCA2-mutant cells may depend on the residual activity of the *SMARCA4*-containing complex, and, vice versa, the knockdown of *SMARCA2* selectively suppresses the growth of SMARCA4-deficient cells. Thus, it is possible to target the residual SWI/SNF complexes by blocking proteins of various subunits to achieve an antitumor effect [224].

The second strategy for obtaining the synthetic lethality of tumor cells is to target the Polycomb repressive complex (PRC2), whose role is opposite to that of the SWI/SNF complex. PRC2 contains a histone methyltransferase that imposes a repressive mark by trimethylating the lysine of histone H3 at the position 27 (H3K27me3), and thus leads to the formation of an inactive chromatin conformation. Inhibition of the catalytic subunit of PRC2 EZH2 has been proposed for the synthetic lethality of tumors with mutations of the SWI/SNF genes [225]. An investigation of five chemical EZH2 inhibitors in cell cultures with mutated *PBRM1* revealed the compound L501-1669, which selectively inhibited the proliferation of cells with a lack of PBRM1, and suppressed the trimethylation of H3K27. At the same time, an increase in apoptotic activity was noted in cells with lack of PBRM1, which contributes to their lethality [226]. It was also demonstrated that EZH2 inhibitors could reduce the viability of ARID1A-deficient cells in a dose-dependent manner in patients with gastric cancer. Confirmation of selective sensitivity to ARID1A-deficient cells in vitro suggests the potential effectiveness of targeted therapy with EZH2 inhibitors for tumors with somatic mutations in *ARID1A* [227].

The SWI/SNF complex subunits ARID1A, ARID1B, and ARID2 are actively involved in the repair of DNA damage, double-stranded breaks (DSB), and non-homologous end junctions (NHEJ) [228]. *ARID1A* mutations prevent the repair of DNA damage in several ways. ARID1A is required to establish open chromatin in DNA damage, which is necessary for the normal functioning of the NHEJ mechanism. The inability of ARID1A mutant cells to repair NHEJ leads to the development of a partial cytotoxic reaction in irradiated cells. The use of irradiation in combination with PARP inhibitors in model mice deficient for ARID1A acts synergistically, enhancing cytotoxicity in ARID1A-negative tumor cells [229]. In addition, the subunits of SWI/SNF complexes participate in DNA damage repair, being located at the sites of double-stranded DNA breaks, and facilitate the phosphorylation of histone H2AX via ATM/ATR [230]. Therefore, tumors with mutations in the genes of the SWI/SNF complex are sensitive to treatment with chemotherapeutic drugs that contribute to DNA damage. Using the participation of genes in DNA repair mechanisms, ARID1A deficiency increases the sensitivity of cells to PARP inhibitors both in vitro and in vivo [231].

It was demonstrated that defects in the PBAF-specific subunit (PBRM1) can also contribute to the state of synthetic lethality of tumor cells when using PARP inhibitors. The mechanism of this sensitivity is associated with the accumulation of R-loops and the replicative stress that occurs during cell division. R-loops are three-stranded structures of nucleic acids that arise during replication and transcription when RNA interacts with double-stranded DNA in the chromatin structure, forming an RNA:DNA hybrid. Their accumulation is also associated with an increased level of DNA damage, especially under the replicative stress. In these conditions, the replication fork stops due to the accumulation of single-stranded breaks (SSBs) and the appearance of abnormal structures (crosslinking or modified bases) in the DNA regions where replication occurs. A higher load on R-loops was noted in tumor cells with PBRM1 deficiency, which contributes to increased replicative stress and DNA damage. Exposure to PARP inhibitors in the presence of a PBRM1 defect further promotes the replication stress, contributing to the accumulation of DNA damage and formation of micronuclei, which leads to the lethality of tumor cells [232].

Since PARP1/2 are enzymes that facilitate SSB repair, excisional base repair, and homologous recombination, their additional inactivation in tumor cells allows, effectively, the blocking of repair mechanisms in addition to the repairing of defects, resulting from mutations in the subunits of chromatin remodeling complexes, and contributes to the development of a synthetic lethality of tumor cells [233].

One more mechanism referring to the genes of the SWI/SNF complex is a modulation of DNA mismatch repair (MMR), which is directly related to an increased mutational load and microsatellite instability. ARID1A interacts with the MMR protein MSH2, functionally regulating its presence at the sites of mismatch of DNA bases without affecting its expression, and leads to increased mutational load and subsequent immunogenicity. The subunits of SWI/SNF complexes also regulate the expression of interferon-sensitive genes (IFN-sensitive genes), limiting the chromatin availability of the EZH2 and PRC2 complexes for interferon-responsive genes [19]. ARID1A aberrations weaken the expression of IFN-dependent genes and the expression of Th1-type chemokines (CXCL9 and CXCL10). SMARCB1 and SMARCA4 modulate the expression of IFN-sensitive genes through interaction with MYC and MAX proteins, respectively, blocking their inhibitory function against IFN-sensitive genes. SMARCB1 directly interacts with MYC through MYC HLH-LZ and SMARCB1 Rpt motifs, whereas SMARCA4 regulates MAX, the functional partner of MYC [19]. These mechanisms demonstrate the importance of using the mutational status of *ARID1A*, *SMARCB1*, and *SMARCA4* as a marker of microsatellite instability and sensitivity to checkpoint inhibitor therapy. Today, all the mechanisms by which chromatin remodeling complexes affect antitumor immunity are not completely described, but it is known that the loss of PBRM1 and ARID2 leads to the increased expression of genes that play a role in the transmission of IFNy (interferon-gamma) signals, which can enhance the response to immunotherapy [234]. It could be explained by the fact that IFNy overexpression activates Janus kinase (JAK) and transcription activator (STAT), which transmit signals affecting all aspects of the immune system, including triggering PD-L1 expression [235]. In addition, it is known that SMARCB1-mutant rhabdoid tumors detect infiltration by subpopulations of T cells, which indicates a tumor-specific immune response [236]. As well as the lack of ARID1A, its interaction with the MMR protein MSH2 contributes to an increase in the tumor mutational load with the subsequent activation of antitumor immunity [237].

It was also revealed that SWI/SNF and PRC2 complexes are directly involved in the control of PD-L1 transcription. In this case, the BRM-containing SWI/SNF complex can act as a transcription repressor of the PD-L1 locus, and the SMARCA4-containing SWI/SNF and PRC2 can jointly activate PD-L1 expression. With mutations and the loss of PBRM1, the remaining SMARCA4 complex interacts with PRC2, leading to changes in chromatin density and a change in the position of the H3K23me3 repression label, although its mechanism remains unclear. These data suggest that targeted epigenetic drugs that inhibit EZH2 can be used as immunomodulators in cancer treatment [238]. Currently, some immune checkpoint inhibitors are being investigated for the treatment of patients with SWI/SNF subunit damage: nivolumab, a fully human antibody IgG4 to PD-1; pembrolizumab (i.e., MK-3475 or lambrolizumab), a highly affinity humanized monoclonal antibody IgG4 targeting PD-1; and MPDL3280A, an engineered antibody IgG against PD-L1 [239].

NcRNAs are also considered as possible therapeutic targets in a wide range of diseases, including cancer. For the last decade, many investigations have been aiming at providing the clinical application of RNA-based therapeutics, and some of them were approved by the FDA. However, the success remains controversial, because of many limitations and challenges. The strategies of therapeutic alteration of ncRNA expression depends on their target genes and functions, and include the suppression of aberrantly overexpressed transcripts, restoration of abnormally downregulated transcripts, and inhibition of their interaction with targets [48,240].

There are several types of RNA-targeting therapeutics. The common therapeutic approaches for miRNA suppression include using chemically modified anti-miRNA oligonucleotides (antimiRs) or sponges, with multiple binding sites to the miRNA of interest. AntimiRs are antisense oligonucleotides (ASOs) with full or partial complementarity to the specific mature miRNA to prevent its interaction with target genes [241]. The miR-122 antimiR Miravirsen (SPC3649; β-D-oxy-LNA) has been clinically tested as a novel hepatitis C virus therapeutic agent [242]. Another anti-miR-92a (MRG-110) has been tested as capable to induce angiogenesis and improve wound healing [243]. MiRNA sponges are artificial transcripts that prevent functioning of specific or different miRNAs by binding them in multiple binding sites [244,245]. For example, a model system was constructed to target miR-21, miR-155, and miR-221/222 that are described as oncogenic miRNAs in multiple tumors [245]. Conversely, synthetic double-stranded miRNA mimics are complementary to the mRNA targets of miRNAs and can restore their expression [5]. Such miRNA mimics have the same sequence as an endogenous miRNA, while the passenger strand carries mismatches to prevent RISC loading and potential action as an antimiR [246]. The miR-34 mimic MRX34 and the miR-16 mimic MesomiR-1 were included in clinical trials for cancer treatment [247,248]. The strategies to decrease the lncRNA level for therapeutic aims include their knockdown by siRNAs or small hairpin RNA (shRNA) via RNA interference or use of ASOs. Upon interacting with target lncRNA, they form an RNA/DNA heteroduplex, which is further cleaved by endogenous RNaseH1 [48].

Eleven RNA-based therapeutics have been approved by the FDA and/or the European Medicines Agency (EMA) so far, while many other candidates undergo phase II or III clinical trials. Among the approved drugs are siRNAs or ASOs that cause specific gene downregulation (e.g., Lumasiran, Fomivirsen) and ASOs that target pre-mRNA splicing (e.g., Viltolarsen, Golodirsen), but no lncRNA-based therapeutics have entered clinical trials yet [246].

The main advantages of miRNA-based therapeutics are their ability to target multiple genes within one pathway, thereby causing a broad but specific response, and their endogenous expression in human cells, which means that all the mechanisms for their processing and downstream target selection already exist. The diversity of mechanisms and functions of lncRNAs also provide numerous opportunities for their therapeutic targeting. However, the limitations of non-coding transcripts are also related to the same characteristics. The ability to target many transcripts in different cells results in the problem of specificity. Thus, the uptake in cells other than the cells of interest may lead to undesired on-target effects, whereas sequence similarities and overdosing to levels much higher than expected endogenously may cause off-target effects [246].

The other important challenge is the in vivo RNA therapeutic delivery to the target tissue. RNA therapeutics are commonly unstable and unable to cross cell membranes because of their negative charge; therefore, chemical modifications are applied to improve their pharmacodynamics and pharmacokinetic properties. There is the replacement of phosphodiester with phosphothiorate backbone linkages, replacement of the 2′-O-alkyl group of the sugar moieties with 2′-O-Me, 2′-MOE or 2′-F to improve bioavailability, enhance efficacy, and reduce toxicity, and the creation of locked nucleic acids (LNAs), phosphoramidate morpholino oligomers (PMOs), or peptide nucleic acids (PNAs) [246,249]. An additional challenge in the therapeutic targeting of lncRNAs is the in vivo validation of such drugs. LncRNAs are poorly conserved across species, and therefore human lncRNAs with few exceptions could not be found in mice. Engineered mouse models with larger human genome segments or entire chromosomes could be an option for such experiments; however, currently it is a rather theoretical than practical option [240].

An Increasing number of epigenetic drugs have been developed in the last decade, and it seems that the combination of two or more therapies toward epigenetic events and/or with other therapies, targeting different signaling pathways, are the most prominent strategy. As modulators of gene expression programs, chromatin proteins can also effectively be targeted in combination therapies. For example, the combination of PARP inhibitor Veliparib and HDAC inhibitor SAHA synergistically caused synthetic lethality in prostate cancer cells [250], whereas the combination of PARP inhibitor Niraparib, HDAC inhibitor Romidepsin or Panobinostat, and a hypomethylating agent Decitabine induced apoptosis in human leukemia and lymphoma cells through trapping PARP1 and DNMT1 onto the chromatin, enhancing the acetylation of DNA repair proteins and downregulation of NuRD [251]. Combination with chemotherapy, radiotherapy, or immunotherapy can also be applied for RNA therapeutics to reduce their doses and prevent adverse effects. This combinatorial therapy could also help with the problem of drug resistance, as many ncRNAs are involved in its regulation [246]. Thus, miR-34a mimics sensitized lung cancer cells to EGFR-specific tyrosine kinase inhibitor erlotinib and radiation [252,253]. Among such miRNAs are several regulators of chromatin remodelers that also may be potential candidates. For example, miR-144-3p that targets ARID1A promotes sunitinib resistance in clear cell renal cell carcinoma [94]; miR-30a-5p that directly targets CHD1 enhances cisplatin sensitivity of ovarian cancer cells through the Wnt/β-catenin pathway [172], whereas lncRNAs uc.57* is involved in BCL11A regulation and thus promotes tamoxifen resistance in breast cancer [129]. These novel epigenetic drugs and their promising therapeutic successes could further lead to progress in the treatment of various diseases, especially cancer.

## 6. Conclusions

Epigenetic regulators affect gene expression without alterations in the nucleotide sequence, and chromatin remodeling is the one of the main mechanisms of epigenetic regulation. Changes in chromatin state are reversible and have a critical role in fundamental biological processes. Many diseases are caused by mutations and other aberrations in chromatin regulators, including chromatin remodeling complexes, DNA and histone modifications, and expression of ncRNAs. Nevertheless, a comprehensive picture of epigenetic patterns both in normal cells and in different diseases is still emerging, and an understanding of the mechanisms underlying their functioning remains challenging. Some of them have already been described; however, because of the multiplicity and variability of epigenetic regulators, even more remains unknown. In addition, it has already become clear that epigenetic regulators are not operating alone, but are tightly connected and form a comprehensive network of regulatory pathways and feedback loops. Chromatin-regulating complexes and ncRNAs that are in focus of our review were also confirmed to regulate each other by complicated and often still unknown mechanisms.

The deregulation of epigenetic mechanisms was observed at the early stages of numerous diseases. Their early appearance and flexibility make them a prominent target for diagnostics, prognosis, and therapy. It is of particular importance for the early diagnosis and therapy of different types of cancer. Currently, it has become possible to use epigenetic machinery as targets for antitumor drugs. In search of effective methods of tumor treatment, all known epigenetic mechanisms are being investigated. These include DNA methylation/demethylation, chemical modification of histone proteins (acetylation, methylation, phosphorylation, etc.), ncRNA, and chromatin remodeling. Some of such epi-drugs and biomarkers are in the stage of clinical trials or have been approved by the FDA for use in clinical practice. Chromatin remodeling complexes are a tempting target for antitumor effects since they have a high frequency of mutations in their subunits.

Since each tumor has its own genetic and epigenetic profile, there is an urgent need to create novel drugs and personalized methods for the effective treatment of patients. Today, the choice of an effective drug for a patient largely depends on a specific set of molecular changes in tumor tissue, and therapeutic strategies are evaluated individually. However, the change in chromatin conformation is a complex process, combining various epigenetic mechanisms, and the subunits of the complex are involved in the implementation of other significant cellular processes. The results of the conducted studies confirm that any normal or tumor cell depends on maintaining a certain chromatin conformation, and it definitely dies when the function regulating the state of chromatin is blocked. As the regulation of the chromatin state is impossible without non-coding transcripts, such as lncRNAs and miRNAs, their significance for the implementation of this complex process is also actively being studied. Pharmacological agents capable of blocking the role of these intermediaries and regulators in the formation of chromatin structure and gene expression are already being developed and investigated.

An increasing number of epigenetic drugs have been developed in the last decade. The combination of two or more therapies to target epigenetic events and/or other therapies targeting different signaling pathways is the most prominent strategy.

Targeting epigenetic regulators for therapy is actively investigated in various phases of clinical trials, which results in an increasing number of novel epigenetic drugs. Their effects on epigenetic machinery in combination with additional chemotherapy, immunotherapy, or targeted therapy can achieve promising results, both in experiments on cell and animal models, and in a clinical setting. Therefore, further understanding of how chromatin remodelers and ncRNAs function in pathological processes and exploring their pharmaceutical potential will lead to more efficient therapies.

## Data Availability

The data presented in the review are openly available in publicly accessible repositories.

## References

[1] Manelyte L. (2017). Chromatin Remodelers, Their Implication in Cancer and Therapeutic Potential. J. Rare Dis. Res. Treat..

[2] Weaver I.C.G., Korgan A.C., Lee K., Wheeler R.V., Hundert A.S., Goguen D. (2017). Stress and the Emerging Roles of Chromatin Remodeling in Signal Integration and Stable Transmission of Reversible Phenotypes. Front. Behav. Neurosci..

[3] Clapier C.R., Cairns B.R. (2009). The Biology of Chromatin Remodeling Complexes. Annu. Rev. Biochem..

[4] Reyes A.A., Marcum R.D., He Y. (2021). Structure and Function of Chromatin Remodelers. J. Mol. Biol..

[5] Bure I.V., Nemtsova M.V., Kuznetsova E.B. (2022). Histone Modifications and Non-Coding RNAs: Mutual Epigenetic Regulation and Role in Pathogenesis. Int. J. Mol. Sci..

[6] Han P., Chang C.-P. (2015). Long Non-Coding RNA and Chromatin Remodeling. RNA Biol..

[7] Tang Y., Wang J., Lian Y., Fan C., Zhang P., Wu Y., Li X., Xiong F., Li X., Li G. (2017). Linking Long Non-Coding RNAs and SWI/SNF Complexes to Chromatin Remodeling in Cancer. Mol. Cancer.

[8] Arif K.M.T., Elliott E.K., Haupt L.M., Griffiths L.R. (2020). Regulatory Mechanisms of Epigenetic MiRNA Relationships in Human Cancer and Potential as Therapeutic Targets. Cancers.

[9] Mirabella A.C., Foster B.M., Bartke T. (2016). Chromatin Deregulation in Disease. Chromosoma.

[10] Längst G., Manelyte L. (2015). Chromatin Remodelers: From Function to Dysfunction. Genes.

[11] Mossink B., Negwer M., Schubert D., Nadif Kasri N. (2021). The Emerging Role of Chromatin Remodelers in Neurodevelopmental Disorders: A Developmental Perspective. Cell. Mol. Life Sci..

[12] Hohmann A.F., Vakoc C.R. (2014). A Rationale to Target the SWI/SNF Complex for Cancer Therapy. Trends Genet..

[13] Krishnamurthy N., Kato S., Lippman S., Kurzrock R. (2022). Chromatin Remodeling (SWI/SNF) Complexes, Cancer, and Response to Immunotherapy. J. Immunother. Cancer.

[14] Wanior M., Krämer A., Knapp S., Joerger A.C. (2021). Exploiting Vulnerabilities of SWI/SNF Chromatin Remodelling Complexes for Cancer Therapy. Oncogene.

[15] Smith-Roe S.L., Nakamura J., Holley D., Chastain P.D., Rosson G.B., Simpson D.A., Ridpath J.R., Kaufman D.G., Kaufmann W.K., Bultman S.J. (2015). SWI/SNF Complexes Are Required for Full Activation of the DNA-Damage Response. Oncotarget.

[16] Neve B., Jonckheere N., Vincent A., Van Seuningen I. (2021). Long Non-Coding RNAs: The Tentacles of Chromatin Remodeler Complexes. Cell. Mol. Life Sci..

[17] Morel D., Almouzni G., Soria J.-C., Postel-Vinay S. (2017). Targeting Chromatin Defects in Selected Solid Tumors Based on Oncogene Addiction, Synthetic Lethality and Epigenetic Antagonism. Ann. Oncol..

[18] Sasaki M., Ogiwara H. (2020). Synthetic Lethal Therapy Based on Targeting the Vulnerability of SWI/SNF Chromatin Remodeling Complex-deficient Cancers. Cancer Sci..

[19] Zhou M., Yuan J., Deng Y., Fan X., Shen J. (2021). Emerging Role of SWI/SNF Complex Deficiency as a Target of Immune Checkpoint Blockade in Human Cancers. Oncogenesis.

[20] Bosse T., ter Haar N.T., Seeber L.M., Diest P.J.v., Hes F.J., Vasen H.F., Nout R.A., Creutzberg C.L., Morreau H., Smit V.T. (2013). Loss of ARID1A Expression and Its Relationship with PI3K-Akt Pathway Alterations, TP53 and Microsatellite Instability in Endometrial Cancer. Mod. Pathol..

[21] Samartzis E.P., Gutsche K., Dedes K.J., Fink D., Stucki M., Imesch P. (2014). Loss of ARID1A Expression Sensitizes Cancer Cells to PI3K- and AKT-Inhibition. Oncotarget.

[22] Kadoch C., Crabtree G.R. (2013). Reversible Disruption of MSWI/SNF (BAF) Complexes by the SS18-SSX Oncogenic Fusion in Synovial Sarcoma. Cell.

[23] Sousa S.B., Hennekam R.C. (2014). The Nicolaides-Baraitser Syndrome. Am. J. Med. Genet..

[24] Tsurusaki Y., Okamoto N., Ohashi H., Kosho T., Imai Y., Hibi-Ko Y., Kaname T., Naritomi K., Kawame H., Wakui K. (2012). Mutations Affecting Components of the SWI/SNF Complex Cause Coffin-Siris Syndrome. Nat. Genet..

[25] Bramswig N.C., Caluseriu O., Lüdecke H.-J., Bolduc F.V., Noel N.C.L., Wieland T., Surowy H.M., Christen H.-J., Engels H., Strom T.M. (2017). Heterozygosity for ARID2 Loss-of-Function Mutations in Individuals with a Coffin–Siris Syndrome-like Phenotype. Hum. Genet..

[26] Li Y., Gong H., Wang P., Zhu Y., Peng H., Cui Y., Li H., Liu J., Wang Z. (2021). The Emerging Role of ISWI Chromatin Remodeling Complexes in Cancer. J. Exp. Clin. Cancer Res..

[27] Aydin Ö.Z., Vermeulen W., Lans H. (2014). ISWI Chromatin Remodeling Complexes in the DNA Damage Response. Cell Cycle.

[28] Erdel F., Schubert T., Marth C., Längst G., Rippe K. (2010). Human ISWI Chromatin-Remodeling Complexes Sample Nucleosomes via Transient Binding Reactions and Become Immobilized at Active Sites. Proc. Natl. Acad. Sci. USA.

[29] Buganim Y., Goldstein I., Lipson D., Milyavsky M., Polak-Charcon S., Mardoukh C., Solomon H., Kalo E., Madar S., Brosh R. (2010). A Novel Translocation Breakpoint within the BPTF Gene Is Associated with a Pre-Malignant Phenotype. PLoS ONE.

[30] Dar A.A., Nosrati M., Bezrookove V., de Semir D., Majid S., Thummala S., Sun V., Tong S., Leong S.P.L., Minor D. (2015). The Role of BPTF in Melanoma Progression and in Response to BRAF-Targeted Therapy. JNCI J. Natl. Cancer Inst..

[31] Stopka T., Zakova D., Fuchs O., Kubrova O., Blafkova J., Jelinek J., Necas E., Zivny J. (2000). Chromatin Remodeling Gene SMARCA5 Is Dysregulated in Primitive Hematopoietic Cells of Acute Leukemia. Leukemia.

[32] Jin Q., Mao X., Li B., Guan S., Yao F., Jin F. (2015). Overexpression of SMARCA5 Correlates with Cell Proliferation and Migration in Breast Cancer. Tumor Biol..

[33] Micucci J.A., Sperry E.D., Martin D.M. (2015). Chromodomain Helicase DNA-Binding Proteins in Stem Cells and Human Developmental Diseases. Stem Cells Dev..

[34] De Majo F., Calore M. (2018). Chromatin Remodelling and Epigenetic State Regulation by Non-Coding RNAs in the Diseased Heart. Non-Coding RNA Res..

[35] Murawska M., Brehm A. (2011). CHD Chromatin Remodelers and the Transcription Cycle. Transcription.

[36] Hoffmann A., Spengler D. (2019). Chromatin Remodeling Complex NuRD in Neurodevelopment and Neurodevelopmental Disorders. Front. Genet..

[37] Sinha S., Molla S., Kundu C.N. (2021). PARP1-Modulated Chromatin Remodeling Is a New Target for Cancer Treatment. Med. Oncol..

[38] Balow S.A., Pierce L.X., Zentner G.E., Conrad P.A., Davis S., Sabaawy H.E., McDermott B.M., Scacheri P.C. (2013). Knockdown of Fbxl10/Kdm2bb Rescues Chd7 Morphant Phenotype in a Zebrafish Model of CHARGE Syndrome. Dev. Biol..

[39] Drivas T.G., Li D., Nair D., Alaimo J.T., Alders M., Altmüller J., Barakat T.S., Bebin E.M., Bertsch N.L., Blackburn P.R. (2020). A Second Cohort of CHD3 Patients Expands the Molecular Mechanisms Known to Cause Snijders Blok-Campeau Syndrome. Eur. J. Hum. Genet..

[40] Bernier R., Golzio C., Xiong B., Stessman H.A., Coe B.P., Penn O., Witherspoon K., Gerdts J., Baker C., Vulto-van Silfhout A.T. (2014). Disruptive CHD8 Mutations Define a Subtype of Autism Early in Development. Cell.

[41] Zlatanova J., Thakar A. (2008). H2A.Z: View from the Top. Structure.

[42] Alatwi H.E., Downs J.A. (2015). Removal of H2A.Z by INO80 Promotes Homologous Recombination. EMBO Rep..

[43] Guo D., Duan X.-Y., Regalado E.S., Mellor-Crummey L., Kwartler C.S., Kim D., Lieberman K., de Vries B.B.A., Pfundt R., Schinzel A. (2017). Loss-of-Function Mutations in YY1AP1 Lead to Grange Syndrome and a Fibromuscular Dysplasia-Like Vascular Disease. Am. J. Hum. Genet..

[44] Kaur J., Daoud A., Eblen S.T. (2019). Targeting Chromatin Remodeling for Cancer Therapy. CMP.

[45] Awan H.M., Shah A., Rashid F., Shan G. (2017). Primate-Specific Long Non-Coding RNAs and MicroRNAs. Genom. Proteom. Bioinform..

[46] Ratti M., Lampis A., Ghidini M., Salati M., Mirchev M.B., Valeri N., Hahne J.C. (2020). MicroRNAs (MiRNAs) and Long Non-Coding RNAs (LncRNAs) as New Tools for Cancer Therapy: First Steps from Bench to Bedside. Targ. Oncol..

[47] Karlsson O., Baccarelli A.A. (2016). Environmental Health and Long Non-Coding RNAs. Curr. Environ. Health Rep..

[48] Bure I.V., Nemtsova M.V. (2021). Methylation and Noncoding RNAs in Gastric Cancer: Everything Is Connected. Int. J. Mol. Sci..

[49] Wei J.-W., Huang K., Yang C., Kang C.-S. (2017). Non-Coding RNAs as Regulators in Epigenetics. Oncol. Rep..

[50] Kozomara A., Birgaoanu M., Griffiths-Jones S. (2019). MiRBase: From MicroRNA Sequences to Function. Nucleic Acids Res..

[51] O’Brien J., Hayder H., Zayed Y., Peng C. (2018). Overview of MicroRNA Biogenesis, Mechanisms of Actions, and Circulation. Front. Endocrinol..

[52] Pajares M.J., Alemany-Cosme E., Goñi S., Bandres E., Palanca-Ballester C., Sandoval J. (2021). Epigenetic Regulation of MicroRNAs in Cancer: Shortening the Distance from Bench to Bedside. Int. J. Mol. Sci..

[53] Ørom U.A., Nielsen F.C., Lund A.H. (2008). MicroRNA-10a Binds the 5′UTR of Ribosomal Protein MRNAs and Enhances Their Translation. Mol. Cell.

[54] Valeri N., Gasparini P., Fabbri M., Braconi C., Veronese A., Lovat F., Adair B., Vannini I., Fanini F., Bottoni A. (2010). Modulation of Mismatch Repair and Genomic Stability by MiR-155. Proc. Natl. Acad. Sci. USA.

[55] Poddar S., Kesharwani D., Datta M. (2017). Interplay between the MiRNome and the Epigenetic Machinery: Implications in Health and Disease. J. Cell. Physiol..

[56] Ali Syeda Z., Langden S.S.S., Munkhzul C., Lee M., Song S.J. (2020). Regulatory Mechanism of MicroRNA Expression in Cancer. Int. J. Mol. Sci..

[57] Zhao L., Wang J., Li Y., Song T., Wu Y., Fang S., Bu D., Li H., Sun L., Pei D. (2021). NONCODEV6: An Updated Database Dedicated to Long Non-Coding RNA Annotation in Both Animals and Plants. Nucleic Acids Res..

[58] Bure I.V., Kuznetsova E.B., Zaletaev D.V. (2018). Long Noncoding RNAs and Their Role in Oncogenesis. Mol. Biol..

[59] Gao N., Li Y., Li J., Gao Z., Yang Z., Li Y., Liu H., Fan T. (2020). Long Non-Coding RNAs: The Regulatory Mechanisms, Research Strategies, and Future Directions in Cancers. Front. Oncol..

[60] Gong L., Zhou X., Sun J. (2021). Circular RNAs Interaction with MiRNAs: Emerging Roles in Breast Cancer. Int. J. Med. Sci..

[61] Clark M.B., Mattick J.S. (2011). Long Noncoding RNAs in Cell Biology. Semin. Cell Dev. Biol..

[62] Guenzl P.M., Barlow D.P. (2012). Macro LncRNAs: A New Layer of *Cis*-Regulatory Information in the Mammalian Genome. RNA Biol..

[63] Cheetham S.W., Gruhl F., Mattick J.S., Dinger M.E. (2013). Long Noncoding RNAs and the Genetics of Cancer. Br. J. Cancer.

[64] Huang V., Li L.-C. (2012). MiRNA Goes Nuclear. RNA Biol..

[65] Place R.F., Li L.-C., Pookot D., Noonan E.J., Dahiya R. (2008). MicroRNA-373 Induces Expression of Genes with Complementary Promoter Sequences. Proc. Natl. Acad. Sci. USA.

[66] Begolli R., Sideris N., Giakountis A. (2019). LncRNAs as Chromatin Regulators in Cancer: From Molecular Function to Clinical Potential. Cancers.

[67] Bayoumi A., Sayed A., Broskova Z., Teoh J.-P., Wilson J., Su H., Tang Y.-L., Kim I. (2016). Crosstalk between Long Noncoding RNAs and MicroRNAs in Health and Disease. Int. J. Mol. Sci..

[68] López-Urrutia E., Bustamante Montes L.P., Ladrón de Guevara Cervantes D., Pérez-Plasencia C., Campos-Parra A.D. (2019). Crosstalk Between Long Non-Coding RNAs, Micro-RNAs and MRNAs: Deciphering Molecular Mechanisms of Master Regulators in Cancer. Front. Oncol..

[69] Rong D., Sun H., Li Z., Liu S., Dong C., Fu K., Tang W., Cao H. (2017). An Emerging Function of CircRNA-MiRNAs-MRNA Axis in Human Diseases. Oncotarget.

[70] Shi Y., Gao S., Zheng Y., Yao M., Ruan F. (2019). LncRNA CASC15 Functions As An Unfavorable Predictor Of Ovarian Cancer Prognosis And Inhibits Tumor Progression Through Regulation Of MiR-221/ARID1A Axis. OTT.

[71] Lei J.-J., Li H.-Q., Mo Z.-H., Liu K.-J., Zhu L.-J., Li C.-Y., Chen W.-L., Zhang L. (2019). Long Noncoding RNA CDKN2B-AS1 Interacts with Transcription Factor BCL11A to Regulate Progression of Cerebral Infarction through Mediating MAP4K1 Transcription. FASEB J..

[72] Xie D., Zhang S., Jiang X., Huang W., He Y., Li Y., Chen S., Xiong H. (2023). Circ_CSPP1 Regulates the Development of Non-Small Cell Lung Cancer via the MiR-486-3p/BRD9 Axis. Biochem. Genet..

[73] Chen B., Wei W., Huang X., Xie X., Kong Y., Dai D., Yang L., Wang J., Tang H., Xie X. (2018). CircEPSTI1 as a Prognostic Marker and Mediator of Triple-Negative Breast Cancer Progression. Theranostics.

[74] Zhou X., Rao Y., Sun Q., Liu Y., Chen J., Bu W. (2019). Long Noncoding RNA CPS1-IT1 Suppresses Melanoma Cell Metastasis through Inhibiting Cyr61 via Competitively Binding to BRG1. J. Cell. Physiol..

[75] Fang C., He W., Xu T., Dai J., Xu L., Sun F. (2019). Upregulation of LncRNA DGCR5 Correlates with Better Prognosis and Inhibits Bladder Cancer Progression via Transcriptionally Facilitating P21 Expression. J. Cell. Physiol..

[76] Wang L.-L., Sun K.-X., Wu D.-D., Xiu Y.-L., Chen X., Chen S., Zong Z.-H., Sang X.-B., Liu Y., Zhao Y. (2017). DLEU1 Contributes to Ovarian Carcinoma Tumourigenesis and Development by Interacting with MiR-490-3p and Altering CDK1 Expression. J. Cell. Mol. Med..

[77] Cajigas I., Leib D.E., Cochrane J., Luo H., Swyter K., Chen S., Clark B.S., Thompson J., Yates J.R., Kingston R.E. (2015). *Evf2 LncRNA* /BRG1/DLX1 Interactions Reveal RNA-Dependent Chromatin Remodeling Inhibition. Development.

[78] Zhang J., Yang Z., Huang Y., Wang K., Xie Y., Yang N. (2021). LncRNA GAS5 Inhibits the Proliferation and Invasion of Ovarian Clear Cell Carcinoma via the miR -31-5p/ARID1A Axis. Kaohsiung J. Med. Sci..

[79] Wang S., Zhang X., Yuan Y., Tan M., Zhang L., Xue X., Yan Y., Han L., Xu Z. (2015). BRG1 Expression Is Increased in Thoracic Aortic Aneurysms and Regulates Proliferation and Apoptosis of Vascular Smooth Muscle Cells through the Long Non-Coding RNA HIF1A-AS1 in Vitro. Eur. J. Cardio-Thorac. Surg..

[80] Imai-Sumida M., Dasgupta P., Kulkarni P., Shiina M., Hashimoto Y., Shahryari V., Majid S., Tanaka Y., Dahiya R., Yamamura S. (2020). Genistein Represses HOTAIR/Chromatin Remodeling Pathways to Suppress Kidney Cancer. Cell. Physiol. Biochem..

[81] Wu H., Wei H., Chen Q. (2020). Long Noncoding RNA HOTTIP Promotes the Metastatic Potential of Ovarian Cancer through the Regulation of the miR -615-3p/SMARCE1 Pathway. Kaohsiung J. Med. Sci..

[82] Liu X., Lu Y., Zhu J., Liu M., Xie M., Ye M., Li M., Wang S., Ming Z., Tong Q. (2019). A Long Noncoding RNA, Antisense IL-7, Promotes Inflammatory Gene Transcription through Facilitating Histone Acetylation and Switch/Sucrose Nonfermentable Chromatin Remodeling. J. Immunol..

[83] Guo X., Wei Y., Wang Z., Liu W., Yang Y., Yu X., He J. (2018). LncRNA LINC00163 Upregulation Suppresses Lung Cancer Development Though Transcriptionally Increasing TCF21 Expression. Am. J. Cancer Res..

[84] Chi F., Qiu F., Jin X., Chen L., He G., Han S. (2022). LINC00982 Inhibits the Proliferation, Migration, and Invasion of Breast Cancer Cells Through the MiR-765/DPF3 Axis. DNA Cell Biol..

[85] Hu G., Gong A.-Y., Wang Y., Ma S., Chen X., Chen J., Su C.-J., Shibata A., Strauss-Soukup J.K., Drescher K.M. (2016). LincRNA-Cox2 Promotes Late Inflammatory Gene Transcription in Macrophages through Modulating SWI/SNF-Mediated Chromatin Remodeling. J. Immunol..

[86] Zhu P., Wang Y., Wu J., Huang G., Liu B., Ye B., Du Y., Gao G., Tian Y., He L. (2016). LncBRM Initiates YAP1 Signalling Activation to Drive Self-Renewal of Liver Cancer Stem Cells. Nat. Commun..

[87] Chen Z., Gao Y., Yao L., Liu Y., Huang L., Yan Z., Zhao W., Zhu P., Weng H. (2018). LncFZD6 Initiates Wnt/β-Catenin and Liver TIC Self-Renewal through BRG1-Mediated FZD6 Transcriptional Activation. Oncogene.

[88] Wang Y., He L., Du Y., Zhu P., Huang G., Luo J., Yan X., Ye B., Li C., Xia P. (2015). The Long Noncoding RNA LncTCF7 Promotes Self-Renewal of Human Liver Cancer Stem Cells through Activation of Wnt Signaling. Cell Stem Cell.

[89] Lino Cardenas C.L., Kessinger C.W., Cheng Y., MacDonald C., MacGillivray T., Ghoshhajra B., Huleihel L., Nuri S., Yeri A.S., Jaffer F.A. (2018). An HDAC9-MALAT1-BRG1 Complex Mediates Smooth Muscle Dysfunction in Thoracic Aortic Aneurysm. Nat. Commun..

[90] Leisegang M.S., Bibli S.-I., Günther S., Pflüger-Müller B., Oo J.A., Höper C., Seredinski S., Yekelchyk M., Schmitz-Rixen T., Schürmann C. (2019). Pleiotropic Effects of Laminar Flow and Statins Depend on the Krüppel-like Factor-Induced LncRNA MANTIS. Eur. Heart J..

[91] Gong X., Huang M.-Y. (2020). Tumor-Suppressive Function of LncRNA-MEG3 in Glioma Cells by Regulating MiR-6088/SMARCB1 Axis. BioMed Res. Int..

[92] Zhang H., Sun Z., Yu L., Sun J. (2017). MiR-139-5p Inhibits Proliferation and Promoted Apoptosis of Human Airway Smooth Muscle Cells by Downregulating the Brg1 Gene. Respir. Physiol. Neurobiol..

[93] Huang H., Wang Y., Li Q., Fei X., Ma H., Hu R. (2019). MiR-140-3p Functions as a Tumor Suppressor in Squamous Cell Lung Cancer by Regulating BRD9. Cancer Lett..

[94] Xiao W., Lou N., Ruan H., Bao L., Xiong Z., Yuan C., Tong J., Xu G., Zhou Y., Qu Y. (2017). Mir-144-3p Promotes Cell Proliferation, Metastasis, Sunitinib Resistance in Clear Cell Renal Cell Carcinoma by Downregulating ARID1A. Cell. Physiol. Biochem..

[95] Li Y., Zhao Y., Cheng M., Qiao Y., Wang Y., Xiong W., Yue W. (2018). Suppression of MicroRNA-144-3p Attenuates Oxygen-Glucose Deprivation/Reoxygenation-Induced Neuronal Injury by Promoting Brg1/Nrf2/ARE Signaling. J. Biochem. Mol. Toxicol..

[96] Li S.-H., Li J.-P., Chen L., Liu J.-L. (2018). MiR-146a Induces Apoptosis in Neuroblastoma Cells by Targeting BCL11A. Med. Hypotheses.

[97] Chang Y., Cui M., Fu X., Zhang L., Li X., Li L., Wu J., Sun Z., Zhang X., Li Z. (2019). MiRNA-155 Regulates Lymphangiogenesis in Natural Killer/T-Cell Lymphoma by Targeting BRG1. Cancer Biol. Ther..

[98] Sakurai K., Furukawa C., Haraguchi T., Inada K., Shiogama K., Tagawa T., Fujita S., Ueno Y., Ogata A., Ito M. (2011). MicroRNAs MiR-199a-5p and -3p Target the Brm Subunit of SWI/SNF to Generate a Double-Negative Feedback Loop in a Variety of Human Cancers. Cancer Res..

[99] Li F., Liang J., Tong H., Zhu S., Tang D. (2020). Inhibition of MicroRNA-199a-5p Ameliorates Oxygen-glucose Deprivation/Reoxygenation-induced Apoptosis and Oxidative Stress in HT22 Neurons by Targeting Brg1 to Activate Nrf2/HO-1 Signalling. Clin. Exp. Pharm. Physiol..

[100] Ke S.-B., Qiu H., Chen J.-M., Shi W., Chen Y.-S. (2018). MicroRNA-202-5p Functions as a Tumor Suppressor in Colorectal Carcinoma by Directly Targeting SMARCC1. Gene.

[101] Schramedei K., Mörbt N., Pfeifer G., Läuter J., Rosolowski M., Tomm J.M., von Bergen M., Horn F., Brocke-Heidrich K. (2011). MicroRNA-21 Targets Tumor Suppressor Genes ANP32A and SMARCA4. Oncogene.

[102] Yang Y., Zhao X., Li H.-X. (2016). MiR-221 and MiR-222 Simultaneously Target ARID1A and Enhance Proliferation and Invasion of Cervical Cancer Cells. Eur. Rev. Med. Pharm. Sci..

[103] Arts F.A., Keogh L., Smyth P., O’Toole S., Ta R., Gleeson N., O’Leary J.J., Flavin R., Sheils O. (2017). MiR-223 Potentially Targets SWI/SNF Complex Protein SMARCD1 in Atypical Proliferative Serous Tumor and High-Grade Ovarian Serous Carcinoma. Hum. Pathol..

[104] Yang F., Xu Y., Liu C., Ma C., Zou S., Xu X., Jia J., Liu Z. (2018). NF-ΚB/MiR-223-3p/ARID1A Axis Is Involved in *Helicobacter pylori* CagA-Induced Gastric Carcinogenesis and Progression. Cell Death Dis..

[105] Zhu Y., Li K., Yan L., He Y., Wang L., Sheng L. (2020). MiR-223-3p Promotes Cell Proliferation and Invasion by Targeting *Arid1a* in Gastric Cancer. ABBS.

[106] Shi D.-M., Shi X.-L., Xing K.-L., Zhou H.-X., Lu L.-L., Wu W.-Z. (2020). MiR-296-5p Suppresses Stem Cell Potency of Hepatocellular Carcinoma Cells via Regulating Brg1/Sall4 Axis. Cell Signal..

[107] Wu H.-J., Zhuo Y., Zhou Y.-C., Wang X.-W., Wang Y.-P., Si C.-Y., Wang X.-H. (2017). MiR-29a Promotes Hepatitis B Virus Replication and Expression by Targeting SMARCE1 in Hepatoma Carcinoma. WJG.

[108] Lu W.-C., Liu C.-J., Tu H.-F., Chung Y.-T., Yang C.-C., Kao S.-Y., Chang K.-W., Lin S.-C. (2016). *MiR-31* Targets ARID1A and Enhances the Oncogenicity and Stemness of Head and Neck Squamous Cell Carcinoma. Oncotarget.

[109] Iwagami Y., Eguchi H., Nagano H., Akita H., Hama N., Wada H., Kawamoto K., Kobayashi S., Tomokuni A., Tomimaru Y. (2013). MiR-320c Regulates Gemcitabine-Resistance in Pancreatic Cancer via SMARCC1. Br. J. Cancer.

[110] Tran N.D., Kissner M., Subramanyam D., Parchem R.J., Laird D.J., Blelloch R.H. (2016). A MiR-372/Let-7 Axis Regulates Human Germ Versus Somatic Cell Fates. Stem Cells.

[111] Lulli V., Romania P., Morsilli O., Cianciulli P., Gabbianelli M., Testa U., Giuliani A., Marziali G. (2013). MicroRNA-486-3p Regulates γ-Globin Expression in Human Erythroid Cells by Directly Modulating BCL11A. PLoS ONE.

[112] Shen J., Xiao Z., Wu W.K.K., Wang M.H., To K.F., Chen Y., Yang W., Li M.S.M., Shin V.Y., Tong J.H. (2015). Epigenetic Silencing of MiR-490-3p Reactivates the Chromatin Remodeler SMARCD1 to Promote *Helicobacter pylori*—Induced Gastric Carcinogenesis. Cancer Res..

[113] Dai J., Lu L., Kang L., Zhang J. (2021). MiR-501-3p Promotes Osteosarcoma Cell Proliferation, Migration and Invasion by Targeting BCL7A. Hum. Cell.

[114] Zhang K., Hu Y., Luo N., Li X., Chen F., Yuan J., Guo L. (2020). MiR-574-5p Attenuates Proliferation, Migration and EMT in Triple-negative Breast Cancer Cells by Targeting BCL11A and SOX2 to Inhibit the SKIL/TAZ/CTGF Axis. Int. J. Oncol..

[115] Sun J., Ye L., Shi Y., Wang X., Zhao X., Ren S., Fan J., Shao H., Qin B. (2022). MiR-6511b-5p Suppresses Metastasis of PMMR Colorectal Cancer through Methylation of CD44 by Directly Targeting BRG1. Clin. Transl. Oncol..

[116] Wang Y., Cao J., Zhang S., Sun L., Nan Y., Yao H., Fan J., Zhu L.Y., Yu L. (2019). MicroRNA-802 Induces Hepatitis B Virus Replication and Replication through Regulating SMARCE1 Expression in Hepatocellular Carcinoma. Cell Death Dis..

[117] Yoo A.S., Staahl B.T., Chen L., Crabtree G.R. (2009). MicroRNA-Mediated Switching of Chromatin-Remodelling Complexes in Neural Development. Nature.

[118] Tamai M., Tatarano S., Okamura S., Fukumoto W., Kawakami I., Osako Y., Sakaguchi T., Sugita S., Yonemori M., Yamada Y. (2022). *MicroRNA-99a-5p* Induces Cellular Senescence in Gemcitabine-resistant Bladder Cancer by Targeting *SMARCD1*. Mol. Oncol..

[119] Cheng S., Wang L., Deng C.-H., Du S.-C., Han Z.-G. (2017). ARID1A Represses Hepatocellular Carcinoma Cell Proliferation and Migration through LncRNA MVIH. Biochem. Biophys. Res. Commun..

[120] Han P., Li W., Lin C.-H., Yang J., Shang C., Nurnberg S.T., Jin K.K., Xu W., Lin C.-Y., Lin C.-J. (2014). A Long Noncoding RNA Protects the Heart from Pathological Hypertrophy. Nature.

[121] Adriaens C., Standaert L., Barra J., Latil M., Verfaillie A., Kalev P., Boeckx B., Wijnhoven P.W.G., Radaelli E., Vermi W. (2016). P53 Induces Formation of NEAT1 LncRNA-Containing Paraspeckles That Modulate Replication Stress Response and Chemosensitivity. Nat. Med..

[122] Han B., Yang M., Liu Q., Wang G., Hou J. (2022). Long Noncoding RNA SBF2-AS1 Promotes Abdominal Aortic Aneurysm Formation through the MiRNA-520f-3p/SMARCD1 Axis. Dis. Mrk..

[123] Raab J.R., Smith K.N., Spear C.C., Manner C.J., Calabrese J.M., Magnuson T. (2019). SWI/SNF Remains Localized to Chromatin in the Presence of SCHLAP1. Nat. Genet..

[124] Prensner J.R., Iyer M.K., Sahu A., Asangani I.A., Cao Q., Patel L., Vergara I.A., Davicioni E., Erho N., Ghadessi M. (2013). The Long Noncoding RNA SChLAP1 Promotes Aggressive Prostate Cancer and Antagonizes the SWI/SNF Complex. Nat. Genet..

[125] Grossi E., Raimondi I., Goñi E., González J., Marchese F.P., Chapaprieta V., Martín-Subero J.I., Guo S., Huarte M. (2020). A LncRNA-SWI/SNF Complex Crosstalk Controls Transcriptional Activation at Specific Promoter Regions. Nat. Commun..

[126] Xia J., Jiang N., Li Y., Wei Y., Zhang X. (2019). The Long Noncoding RNA THRIL Knockdown Protects Hypoxia-Induced Injuries of H9C2 Cells through Regulating MiR-99a. Cardiol. J..

[127] Ming N., Na H.S.T., He J.-L., Meng Q.-T., Xia Z.-Y. (2019). Propofol Alleviates Oxidative Stress via Upregulating LncRNA-TUG1/Brg1 Pathway in Hypoxia/Reoxygenation Hepatic Cells. J. Biochem..

[128] Panatta E., Lena A.M., Mancini M., Smirnov A., Marini A., Delli Ponti R., Botta-Orfila T., Tartaglia G.G., Mauriello A., Zhang X. (2020). Long Non-coding RNA Uc.291 Controls Epithelial Differentiation by Interfering with the ACTL6A/BAF Complex. EMBO Rep..

[129] Zhang C.-H., Wang J., Zhang L.-X., Lu Y.-H., Ji T.-H., Xu L., Ling L.-J. (2017). Shikonin Reduces Tamoxifen Resistance through Long Non-Coding RNA Uc.57. Oncotarget.

[130] Wang X., Gong Y., Jin B., Wu C., Yang J., Wang L., Zhang Z., Mao Z. (2014). Long Non-Coding RNA Urothelial Carcinoma Associated 1 Induces Cell Replication by Inhibiting BRG1 in 5637 Cells. Oncol. Rep..

[131] Jégu T., Blum R., Cochrane J.C., Yang L., Wang C.-Y., Gilles M.-E., Colognori D., Szanto A., Marr S.K., Kingston R.E. (2019). Xist RNA Antagonizes the SWI/SNF Chromatin Remodeler BRG1 on the Inactive X Chromosome. Nat. Struct. Mol. Biol..

[132] Xie Z.-F., Li H.-T., Xie S.-H., Ma M. (2020). Circular RNA Hsa_circ_0006168 Contributes to Cell Proliferation, Migration and Invasion in Esophageal Cancer by Regulating MiR-384/RBBP7 Axis via Activation of S6K/S6 Pathway. Eur. Rev. Med. Pharmacol. Sci..

[133] Cao X., Li F., Shao J., Lv J., Chang A., Dong W., Zhu F. (2021). Circular RNA Hsa_circ_0102231 Sponges MiR-145 to Promote Non-Small Cell Lung Cancer Cell Proliferation by up-Regulating the Expression of RBBP4. J. Biochem..

[134] Ding L., Zhao Y., Dang S., Wang Y., Li X., Yu X., Li Z., Wei J., Liu M., Li G. (2019). Circular RNA Circ-DONSON Facilitates Gastric Cancer Growth and Invasion via NURF Complex Dependent Activation of Transcription Factor SOX4. Mol. Cancer.

[135] Liu T., Han Z., Li H., Zhu Y., Sun Z., Zhu A. (2018). LncRNA DLEU1 Contributes to Colorectal Cancer Progression via Activation of KPNA3. Mol. Cancer.

[136] Li D., Jiang X., Zhang X., Cao G., Wang D., Chen Z. (2019). Long Noncoding RNA FGD5-AS1 Promotes Colorectal Cancer Cell Proliferation, Migration, and Invasion through Upregulating CDCA7 via Sponging MiR-302e. Vitr. Cell. Dev. Biol.-Anim..

[137] Liu Y., Chen J., Zhou L., Yin C. (2022). LINC00885 Promotes Cervical Cancer Progression through Sponging MiR-3150b-3p and Upregulating BAZ2A. Biol. Direct..

[138] Guetg C., Scheifele F., Rosenthal F., Hottiger M.O., Santoro R. (2012). Inheritance of Silent RDNA Chromatin Is Mediated by PARP1 via Noncoding RNA. Mol. Cell.

[139] Ye B., Liu B., Yang L., Zhu X., Zhang D., Wu W., Zhu P., Wang Y., Wang S., Xia P. (2018). *LncKdm2b* Controls Self-renewal of Embryonic Stem Cells via Activating Expression of Transcription Factor *Zbtb3*. EMBO J..

[140] Liu B., Ye B., Yang L., Zhu X., Huang G., Zhu P., Du Y., Wu J., Qin X., Chen R. (2017). Long Noncoding RNA LncKdm2b Is Required for ILC3 Maintenance by Initiation of Zfp292 Expression. Nat. Immunol..

[141] Shou J., Gao H., Cheng S., Wang B., Guan H. (2021). LncRNA HOXA-AS2 Promotes Glioblastoma Carcinogenesis by Targeting MiR-885-5p/RBBP4 Axis. Cancer Cell Int..

[142] Wang H., Tan L., Dong X., Liu L., Jiang Q., Li H., Shi J., Yang X., Dai X., Qian Z. (2020). MiR-146b-5p Suppresses the Malignancy of GSC/MSC Fusion Cells by Targeting SMARCA5. Aging.

[143] Tommasi S., Pinto R., Danza K., Pilato B., Palumbo O., Micale L., Summa S.D. (2016). MiR-151-5p, Targeting Chromatin Remodeler SMARCA5, as a Marker for the BRCAness Phenotype. Oncotarget.

[144] Li L., Tang J., Zhang B., Yang W., LiuGao M., Wang R., Tan Y., Fan J., Chang Y., Fu J. (2015). Epigenetic Modification of MiR-429 Promotes Liver Tumour-Initiating Cell Properties by Targeting Rb Binding Protein 4. Gut.

[145] Li Y., Li J., Luo M., Zhou C., Shi X., Yang W., Lu Z., Chen Z., Sun N., He J. (2018). Novel Long Noncoding RNA NMR Promotes Tumor Progression via NSUN2 and BPTF in Esophageal Squamous Cell Carcinoma. Cancer Lett..

[146] Pavlaki I., Alammari F., Sun B., Clark N., Sirey T., Lee S., Woodcock D.J., Ponting C.P., Szele F.G., Vance K.W. (2018). The Long Non-coding RNA *Paupar* Promotes KAP 1-dependent Chromatin Changes and Regulates Olfactory Bulb Neurogenesis. EMBO J..

[147] Wang C.-H., Li Q.-Y., Nie L., Ma J., Yao C.-J., Chen F.-P. (2020). LncRNA ANRIL Promotes Cell Proliferation, Migration and Invasion during Acute Myeloid Leukemia Pathogenesis via Negatively Regulating MiR-34a. Int. J. Biochem. Cell Biol..

[148] Lu C., Wang X., Zhao X., Xin Y., Liu C. (2020). Long Non-Coding RNA ARAP1-AS1 Accelerates Cell Proliferation and Migration in Breast Cancer through MiR-2110/HDAC2/PLIN1 Axis. Biosci. Rep..

[149] Rom A., Melamed L., Gil N., Goldrich M.J., Kadir R., Golan M., Biton I., Perry R.B.-T., Ulitsky I. (2019). Regulation of CHD2 Expression by the Chaserr Long Noncoding RNA Gene Is Essential for Viability. Nat. Commun..

[150] Hao J., Du X., Lv F., Shi Q. (2021). Knockdown of Circ_0006528 Suppresses Cell Proliferation, Migration, Invasion, and Adriamycin Chemoresistance via Regulating the MiR-1236-3p/CHD4 Axis in Breast Cancer. J. Surg. Res..

[151] Wang Y., Huang X., Wang P., Zeng Y., Zhou G. (2022). The Hsa_circ_0007396-MiR-767-3p-CHD4 Axis Is Involved in the Progression and Carcinogenesis of Gastric Cancer. J. Gastrointest. Oncol..

[152] Yang Y., Ding L., Li Y., Xuan C. (2020). Hsa_circ_0039411 Promotes Tumorigenesis and Progression of Papillary Thyroid Cancer by MiR-1179/ABCA9 and MiR-1205/MTA1 Signaling Pathways. J. Cell. Physiol..

[153] Luan S., Fu P., Wang X., Gao Y., Shi K., Guo Y. (2020). Circular RNA Circ-NCOR2 Accelerates Papillary Thyroid Cancer Progression by Sponging MiR-516a-5p to Upregulate Metastasis-Associated Protein 2 Expression. J. Int. Med. Res..

[154] Xia T., Pan Z., Zhang J. (2020). CircPDZD8 Promotes Gastric Cancer Progression by Regulating CHD9 via Sponging MiR-197-5p. Aging.

[155] Sheng M., Wei N., Yang H.-Y., Yan M., Zhao Q.-X., Jing L.-J. (2019). CircRNA UBAP2 Promotes the Progression of Ovarian Cancer by Sponging MicroRNA-144. Eur. Rev. Med. Pharmacol. Sci..

[156] Wang S., Li Q., Wang Y., Li X., Wang R., Kang Y., Xue X., Meng R., Wei Q., Feng X. (2018). Upregulation of Circ-UBAP2 Predicts Poor Prognosis and Promotes Triple-Negative Breast Cancer Progression through the MiR-661/MTA1 Pathway. Biochem. Biophys. Res. Commun..

[157] He Y., Zhang Z., Wang Z., Jiao Y., Kang Q., Li J. (2022). Downregulation of Circ-SFMBT2 Blocks the Development of Gastric Cancer by Targeting the MiR-885-3p/CHD7 Pathway. Anti-Cancer Drugs..

[158] Zhai L.-Q., Wang X.-X., Qu C.-X., Yang L.-Z., Jia C.-M., Shi X.-C. (2021). A Long Non-Coding RNA, ELFN1−AS1, Sponges MiR-1250 to Upregulate MTA1 to Promote Cell Proliferation, Migration and Invasion, and Induce Apoptosis in Colorectal Cancer. Eur. Rev. Med. Pharmacol. Sci..

[159] Tao D., Zhang Z., Liu X., Zhang Z., Fu Y., Zhang P., Yuan H., Liu L., Cheng J., Jiang H. (2020). LncRNA HOTAIR Promotes the Invasion and Metastasis of Oral Squamous Cell Carcinoma through Metastasis-associated Gene 2. Mol. Carcinog..

[160] Evsen L., Li X., Zhang S., Razin S., Doetzlhofer A. (2020). *Let-7* MiRNAs Inhibit CHD7 Expression and Control Auditory-Sensory Progenitor Cell Behavior in the Developing Inner Ear. Development.

[161] Zhou N., Zhu X., Man L. (2020). LINC00963 Functions as an Oncogene in Bladder Cancer by Regulating the MiR-766-3p/MTA1 Axis. CMAR.

[162] Lu M., Ding N., Zhuang S., Li Y. (2020). LINC01410/MiR-23c/CHD7 Functions as a CeRNA Network to Affect the Prognosis of Patients with Endometrial Cancer and Strengthen the Malignant Properties of Endometrial Cancer Cells. Mol. Cell Biochem..

[163] Li J., Xu J., Zheng S., Cheng S. (2022). LncRNA LINC02535 Induces Colorectal Adenocarcinoma Progression via Modulating MiR-30d-5p/CHD1. Mol. Biotechnol..

[164] Zhu J., Gu W., Yu C. (2020). MATN1-AS1 Promotes Glioma Progression by Functioning as CeRNA of MiR-200b/c/429 to Regulate CHD1 Expression. Cell Prolif.

[165] An J.-X., Ma M.-H., Zhang C.-D., Shao S., Zhou N.-M., Dai D.-Q. (2018). MiR-1236-3p Inhibits Invasion and Metastasis in Gastric Cancer by Targeting MTA2. Cancer Cell Int..

[166] Song D., Zhang Q., Zhang H., Zhan L., Sun X. (2022). MiR-130b-3p Promotes Colorectal Cancer Progression by Targeting CHD9. Cell Cycle.

[167] Yao B., Wan X., Zheng X., Zhong T., Hu J., Zhou Y., Qin A., Ma Y., Yin D. (2020). Critical Roles of MicroRNA-141-3p and CHD8 in Hypoxia/Reoxygenation-Induced Cardiomyocyte Apoptosis. Cell Biosci..

[168] Wu M., Ye X., Wang S., Li Q., Lai Y., Yi Y. (2017). MicroRNA-148b Suppresses Proliferation, Migration, and Invasion of Nasopharyngeal Carcinoma Cells by Targeting Metastasis-Associated Gene 2. OTT.

[169] Yuan H., Du S., Deng Y., Xu X., Zhang Q., Wang M., Wang P., Su Y., Liang X., Sun Y. (2019). Effects of MicroRNA-208a on Inflammation and Oxidative Stress in Ketamine-Induced Cardiotoxicity through Notch/NF-ΚB Signal Pathways by CHD9. Biosci. Rep..

[170] Chen H., Xu X., Liu Z., Wu Y. (2021). MiR-22-3p Suppresses Vascular Remodeling and Oxidative Stress by Targeting CHD9 during the Development of Hypertension. J. Vasc. Res..

[171] Zhao A., Zhao Y., Feng W., Zhao Z., Liu W., Wang N., Xue H., Wu L., Cui S., Bai R. (2022). MiR-30 Inhibits the Progression of Osteosarcoma by Targeting MTA1. J. Musculoskelet. Neuronal. Interact..

[172] Wang X., Zhao H., Wang P., Zhang J., Li N., Liu Y., Zhang F., Yu Y. (2022). MiR-30a-5p/CHD1 Axis Enhances Cisplatin Sensitivity of Ovarian Cancer Cells via Inactivating the Wnt/β-Catenin Pathway. Anti-Cancer Drugs..

[173] Cao J., Li G., Han M., Xu H., Huang K. (2017). MiR-30c-5p Suppresses Migration, Invasion and Epithelial to Mesenchymal Transition of Gastric Cancer via Targeting MTA1. Biomed. Pharmacother..

[174] Xue L., Yang D. (2018). MiR-421 Inhibited Proliferation and Metastasis of Colorectal Cancer by Targeting MTA1. J. BUON.

[175] Pan Y., Liang W., Zhao X., Liu L., Qing Y., Li Y. (2016). MiR-548b Inhibits the Proliferation and Invasion of Malignant Gliomas by Targeting Metastasis Tumor-Associated Protein-2. NeuroReport.

[176] Chu J. (2019). MicroRNA-589 Serves as a Tumor Suppressor MicroRNA through Directly Targeting Metastasis-associated Protein 2 in Breast Cancer. Oncol. Lett..

[177] Zhao Z., Sentürk N., Song C., Grummt I. (2018). LncRNA PAPAS Tethered to the RDNA Enhancer Recruits Hypophosphorylated CHD4/NuRD to Repress RRNA Synthesis at Elevated Temperatures. Genes Dev..

[178] Dai W., Dai J.-L., Tang M.-H., Ye M.-S., Fang S. (2019). LncRNA-SNHG15 Accelerates the Development of Hepatocellular Carcinoma by Targeting MiR-490-3p/ Histone Deacetylase 2 Axis. WJG.

[179] Zhou Z., Yang P., Zhang B., Yao M., Jia Y., Li N., Liu H., Bai H., Gong X. (2021). Long Noncoding RNA TTC39A-AS1 Promotes Breast Cancer Tumorigenicity by Sponging MicroRNA-483-3p and Thereby Upregulating MTA2. Pharmacology.

[180] Zhou X., Han X., Wittfeldt A., Sun J., Liu C., Wang X., Gan L.-M., Cao H., Liang Z. (2016). Long Non-Coding RNA ANRIL Regulates Inflammatory Responses as a Novel Component of NF-ΚB Pathway. RNA Biol..

[181] Zhang Z., Fu C., Xu Q., Wei X. (2017). Long Non-Coding RNA CASC7 Inhibits the Proliferation and Migration of Colon Cancer Cells via Inhibiting MicroRNA-21. Biomed. Pharmacother..

[182] Chen J., Liu G., Wu Y., Ma J., Wu H., Xie Z., Chen S., Yang Y., Wang S., Shen P. (2019). CircMYO10 Promotes Osteosarcoma Progression by Regulating MiR-370-3p/RUVBL1 Axis to Enhance the Transcriptional Activity of β-Catenin/LEF1 Complex via Effects on Chromatin Remodeling. Mol. Cancer.

[183] Chang C., Liu T.-Y., Lee Y., Chen Y.-C., Yeh K., Lee C., Chen Y., Lin P.-C., Chang Y., Chan W. (2018). Genome-Wide Analysis of LncRNAs in 3’-Untranslated Regions: CR933609 Acts as a Decoy to Protect the INO80D Gene. Int. J. Oncol..

[184] Zhang Z., Hu X., Kuang J., Liao J., Yuan Q. (2020). LncRNA DRAIC Inhibits Proliferation and Metastasis of Gastric Cancer Cells through Interfering with NFRKB Deubiquitination Mediated by UCHL5. Cell. Mol. Biol. Lett..

[185] Wang Y., Zhu P., Luo J., Wang J., Liu Z., Wu W., Du Y., Ye B., Wang D., He L. (2019). LncRNA HAND2-AS1 Promotes Liver Cancer Stem Cell Self-renewal via BMP Signaling. EMBO J..

[186] Zhang J., Li N., Fu J., Zhou W. (2020). Long Noncoding RNA HOTAIR Promotes Medulloblastoma Growth, Migration and Invasion by Sponging MiR-1/MiR-206 and Targeting YY1. Biomed. Pharmacother..

[187] Wang B., Wang Y., Ma D., Wang L., Yang M. (2020). Long Noncoding RNA LCTS5 Inhibits Non-Small Cell Lung Cancer by Interacting with INO80. Life Sci..

[188] Xuan W., Zhou C., You G. (2020). LncRNA LINC00668 Promotes Cell Proliferation, Migration, Invasion Ability and EMT Process in Hepatocellular Carcinoma by Targeting MiR-532-5p/YY1 Axis. Biosci. Rep..

[189] Liu X., Chen J., Zhang S., Liu X., Long X., Lan J., Zhou M., Zheng L., Zhou J. (2022). LINC00839 Promotes Colorectal Cancer Progression by Recruiting RUVBL1/Tip60 Complexes to Activate NRF1. EMBO Rep..

[190] Dong X., Xu X., Guan Y. (2020). LncRNA LINC00899 Promotes Progression of Acute Myeloid Leukaemia by Modulating MiR-744-3p/YY1 Signalling. Cell Biochem. Funct..

[191] Rong Z., Wang Z., Wang X., Qin C., Geng W. (2020). Molecular Interplay between Linc01134 and YY1 Dictates Hepatocellular Carcinoma Progression. J. Exp. Clin. Cancer Res..

[192] Schutt C., Hallmann A., Hachim S., Klockner I., Valussi M., Atzberger A., Graumann J., Braun T., Boettger T. (2020). Linc- MYH Configures INO 80 to Regulate Muscle Stem Cell Numbers and Skeletal Muscle Hypertrophy. EMBO J..

[193] Zhou L., Sun K., Zhao Y., Zhang S., Wang X., Li Y., Lu L., Chen X., Chen F., Bao X. (2015). Linc-YY1 Promotes Myogenic Differentiation and Muscle Regeneration through an Interaction with the Transcription Factor YY1. Nat. Commun..

[194] Huang G., Jiang H., Lin Y., Wu Y., Cai W., Shi B., Luo Y., Jian Z., Zhou X. (2018). LncAKHE Enhances Cell Growth and Migration in Hepatocellular Carcinoma via Activation of NOTCH2 Signaling. Cell Death Dis..

[195] Liu M.-X., Zhou K.-C., Cao Y. (2014). MCRS1 Overexpression, Which Is Specifically Inhibited by MiR-129*, Promotes the Epithelial-Mesenchymal Transition and Metastasis in Non-Small Cell Lung Cancer. Mol. Cancer.

[196] Tian C., Deng Y., Jin Y., Shi S., Bi H. (2018). Long Non-Coding RNA RNCR3 Promotes Prostate Cancer Progression through Targeting MiR-185-5p. Am. J. Transl. Res..

[197] Xu J., Deng Y., Wang Y., Sun X., Chen S., Fu G. (2020). SPAG5-AS1 Inhibited Autophagy and Aggravated Apoptosis of Podocytes via SPAG5/AKT/MTOR Pathway. Cell Prolif..

[198] Katsushima K., Natsume A., Ohka F., Shinjo K., Hatanaka A., Ichimura N., Sato S., Takahashi S., Kimura H., Totoki Y. (2016). Targeting the Notch-Regulated Non-Coding RNA TUG1 for Glioma Treatment. Nat. Commun..

[199] Patty B.J., Hainer S.J. (2020). Non-Coding RNAs and Nucleosome Remodeling Complexes: An Intricate Regulatory Relationship. Biology.

[200] Gao C., Zhang J., Wang Q., Ren C. (2016). Overexpression of LncRNA NEAT1 Mitigates Multidrug Resistance by Inhibiting ABCG2 in Leukemia. Oncol. Lett..

[201] Sun C., Li S., Zhang F., Xi Y., Wang L., Bi Y., Li D. (2016). Long Non-Coding RNA NEAT1 Promotes Non-Small Cell Lung Cancer Progression through Regulation of MiR-377-3p-E2F3 Pathway. Oncotarget.

[202] Ke H., Zhao L., Feng X., Xu H., Zou L., Yang Q., Su X., Peng L., Jiao B. (2016). NEAT1 Is Required for Survival of Breast Cancer Cells through FUS and MiR-548. Gene Regul. Syst. Biol..

[203] Fu J.-W., Kong Y., Sun X. (2016). Long Noncoding RNA NEAT1 Is an Unfavorable Prognostic Factor and Regulates Migration and Invasion in Gastric Cancer. J. Cancer Res. Clin. Oncol..

[204] Hainer S.J., Gu W., Carone B.R., Landry B.D., Rando O.J., Mello C.C., Fazzio T.G. (2015). Suppression of Pervasive Noncoding Transcription in Embryonic Stem Cells by EsBAF. Genes Dev..

[205] Chen M., Herring B.P. (2013). Regulation of MicroRNAs by Brahma-Related Gene 1 (Brg1) in Smooth Muscle Cells. J. Biol. Chem..

[206] Wang G., Fu Y., Yang X., Luo X., Wang J., Gong J., Hu J. (2017). Erratum: Brg-1 Targeting of Novel MiR550a-5p/RNF43/Wnt Signaling Axis Regulates Colorectal Cancer Metastasis. Oncogene.

[207] Zhang Z., Li J., Guo H., Wang F., Ma L., Du C., Wang Y., Wang Q., Kornmann M., Tian X. (2019). BRM Transcriptionally Regulates MiR-302a-3p to Target SOCS5/STAT3 Signaling Axis to Potentiate Pancreatic Cancer Metastasis. Cancer Lett..

[208] Hu Y.-W., Guo F.-X., Xu Y.-J., Li P., Lu Z.-F., McVey D.G., Zheng L., Wang Q., Ye J.H., Kang C.-M. (2019). Long Noncoding RNA NEXN-AS1 Mitigates Atherosclerosis by Regulating the Actin-Binding Protein NEXN. J. Clin. Investig..

[209] Kong Z., Wan X., Zhang Y., Zhang P., Zhang Y., Zhang X., Qi X., Wu H., Huang J., Li Y. (2017). Androgen-Responsive Circular RNA CircSMARCA5 Is up-Regulated and Promotes Cell Proliferation in Prostate Cancer. Biochem. Biophys. Res. Commun..

[210] Hennig B.P., Bendrin K., Zhou Y., Fischer T. (2012). Chd1 Chromatin Remodelers Maintain Nucleosome Organization and Repress Cryptic Transcription. EMBO Rep..

[211] Zhao Z., Dammert M.A., Grummt I., Bierhoff H. (2016). LncRNA-Induced Nucleosome Repositioning Reinforces Transcriptional Repression of RRNA Genes upon Hypotonic Stress. Cell Rep..

[212] Schnetz M.P., Bartels C.F., Shastri K., Balasubramanian D., Zentner G.E., Balaji R., Zhang X., Song L., Wang Z., LaFramboise T. (2009). Genomic Distribution of CHD7 on Chromatin Tracks H3K4 Methylation Patterns. Genome Res..

[213] Menon T., Yates J.A., Bochar D.A. (2010). Regulation of Androgen-Responsive Transcription by the Chromatin Remodeling Factor CHD8. Mol. Endocrinol..

[214] Wang X., Liu Y., Fan Y., Liu Z., Yuan Q., Jia M., Geng Z., Gu L., Lu X. (2018). LncRNA PTCSC3 Affects Drug Resistance of Anaplastic Thyroid Cancer through STAT3/INO80 Pathway. Cancer Biol. Ther..

[215] Wang Y., Zhu P., Wang J., Zhu X., Luo J., Meng S., Wu J., Ye B., He L., Du Y. (2018). Long Noncoding RNA LncHand2 Promotes Liver Repopulation via C-Met Signaling. J. Hepatol..

[216] Biegel J.A., Zhou J.Y., Rorke L.B., Stenstrom C., Wainwright L.M., Fogelgren B. (1999). Germ-Line and Acquired Mutations of INI1 in Atypical Teratoid and Rhabdoid Tumors. Cancer Res..

[217] Zhang L., Lu Q., Chang C., Chang C., Lu Q. (2020). Epigenetics in Health and Disease. Epigenetics in Allergy and Autoimmunity.

[218] Cochran A.G., Conery A.R., Sims R.J. (2019). Bromodomains: A New Target Class for Drug Development. Nat. Rev. Drug. Discov..

[219] Vangamudi B., Paul T.A., Shah P.K., Kost-Alimova M., Nottebaum L., Shi X., Zhan Y., Leo E., Mahadeshwar H.S., Protopopov A. (2015). The SMARCA2/4 ATPase Domain Surpasses the Bromodomain as a Drug Target in SWI/SNF-Mutant Cancers: Insights from CDNA Rescue and PFI-3 Inhibitor Studies. Cancer Res..

[220] Xiao L., Parolia A., Qiao Y., Bawa P., Eyunni S., Mannan R., Carson S.E., Chang Y., Wang X., Zhang Y. (2022). Targeting SWI/SNF ATPases in Enhancer-Addicted Prostate Cancer. Nature.

[221] Schick S., Grosche S., Kohl K.E., Drpic D., Jaeger M.G., Marella N.C., Imrichova H., Lin J.-M.G., Hofstätter G., Schuster M. (2021). Acute BAF Perturbation Causes Immediate Changes in Chromatin Accessibility. Nat. Genet..

[222] Iurlaro M., Stadler M.B., Masoni F., Jagani Z., Galli G.G., Schübeler D. (2021). Mammalian SWI/SNF Continuously Restores Local Accessibility to Chromatin. Nat. Genet..

[223] Moe K.C., Maxwell J.N., Wang J., Jones C.A., Csaki G.T., Florian A.C., Romer A.S., Bryant D.L., Farone A.L., Liu Q. (2022). The SWI/SNF ATPase BRG1 Facilitates Multiple pro-Tumorigenic Gene Expression Programs in SMARCB1-Deficient Cancer Cells. Oncogenesis.

[224] Oike T., Ogiwara H., Tominaga Y., Ito K., Ando O., Tsuta K., Mizukami T., Shimada Y., Isomura H., Komachi M. (2013). A Synthetic Lethality–Based Strategy to Treat Cancers Harboring a Genetic Deficiency in the Chromatin Remodeling Factor BRG1. Cancer Res..

[225] Bitler B.G., Aird K.M., Zhang R. (2016). Epigenetic Synthetic Lethality in Ovarian Clear Cell Carcinoma: EZH2 and *ARID1A* Mutations. Mol. Cell. Oncol..

[226] Huang K., Sun R., Chen J., Yang Q., Wang Y., Zhang Y., Xie K., Zhang T., Li R., Zhao Q. (2020). A Novel EZH2 Inhibitor Induces Synthetic Lethality and Apoptosis in PBRM1-Deficient Cancer Cells. Cell Cycle.

[227] Yamada L., Saito M., Thar Min A.K., Saito K., Ashizawa M., Kase K., Nakajima S., Onozawa H., Okayama H., Endo H. (2021). Selective Sensitivity of EZH2 Inhibitors Based on Synthetic Lethality in ARID1A-Deficient Gastric Cancer. Gastric Cancer.

[228] Mathur R. (2018). ARID1A Loss in Cancer: Towards a Mechanistic Understanding. Pharmacol. Ther..

[229] Park Y., Chui M.H., Suryo Rahmanto Y., Yu Z.-C., Shamanna R.A., Bellani M.A., Gaillard S., Ayhan A., Viswanathan A., Seidman M.M. (2019). Loss of ARID1A in Tumor Cells Renders Selective Vulnerability to Combined Ionizing Radiation and PARP Inhibitor Therapy. Clin. Cancer Res..

[230] Park J.-H., Park E.-J., Lee H.-S., Kim S.J., Hur S.-K., Imbalzano A.N., Kwon J. (2006). Mammalian SWI/SNF Complexes Facilitate DNA Double-Strand Break Repair by Promoting γ-H2AX Induction. EMBO J..

[231] Shen J., Peng Y., Wei L., Zhang W., Yang L., Lan L., Kapoor P., Ju Z., Mo Q., Shih I.-M. (2015). ARID1A Deficiency Impairs the DNA Damage Checkpoint and Sensitizes Cells to PARP Inhibitors. Cancer Discov..

[232] Chabanon R.M., Morel D., Eychenne T., Colmet-Daage L., Bajrami I., Dorvault N., Garrido M., Meisenberg C., Lamb A., Ngo C. (2021). PBRM1 Deficiency Confers Synthetic Lethality to DNA Repair Inhibitors in Cancer. Cancer Res..

[233] Tsuda M., Fukuda A., Kawai M., Araki O., Seno H. (2021). The Role of the SWI/SNF Chromatin Remodeling Complex in Pancreatic Ductal Adenocarcinoma. Cancer Sci..

[234] Pan D., Kobayashi A., Jiang P., Ferrari de Andrade L., Tay R.E., Luoma A.M., Tsoucas D., Qiu X., Lim K., Rao P. (2018). A Major Chromatin Regulator Determines Resistance of Tumor Cells to T Cell–Mediated Killing. Science.

[235] Villarino A.V., Kanno Y., O’Shea J.J. (2017). Mechanisms and Consequences of Jak–STAT Signaling in the Immune System. Nat. Immunol..

[236] Leruste A., Tosello J., Ramos R.N., Tauziède-Espariat A., Brohard S., Han Z.-Y., Beccaria K., Andrianteranagna M., Caudana P., Nikolic J. (2019). Clonally Expanded T Cells Reveal Immunogenicity of Rhabdoid Tumors. Cancer Cell.

[237] Shen J., Ju Z., Zhao W., Wang L., Peng Y., Ge Z., Nagel Z.D., Zou J., Wang C., Kapoor P. (2018). ARID1A Deficiency Promotes Mutability and Potentiates Therapeutic Antitumor Immunity Unleashed by Immune Checkpoint Blockade. Nat. Med..

[238] Jancewicz I., Szarkowska J., Konopinski R., Stachowiak M., Swiatek M., Blachnio K., Kubala S., Oksinska P., Cwiek P., Rusetska N. (2021). PD-L1 Overexpression, SWI/SNF Complex Deregulation, and Profound Transcriptomic Changes Characterize Cancer-Dependent Exhaustion of Persistently Activated CD4+ T Cells. Cancers.

[239] Carbognin L., Pilotto S., Milella M., Vaccaro V., Brunelli M., Caliò A., Cuppone F., Sperduti I., Giannarelli D., Chilosi M. (2015). Differential Activity of Nivolumab, Pembrolizumab and MPDL3280A According to the Tumor Expression of Programmed Death-Ligand-1 (PD-L1): Sensitivity Analysis of Trials in Melanoma, Lung and Genitourinary Cancers. PLoS ONE.

[240] Fathi Dizaji B. (2020). Strategies to Target Long Non-Coding RNAs in Cancer Treatment: Progress and Challenges. Egypt. J. Med. Hum. Genet..

[241] Krützfeldt J., Rajewsky N., Braich R., Rajeev K.G., Tuschl T., Manoharan M., Stoffel M. (2005). Silencing of MicroRNAs in Vivo with ‘Antagomirs’. Nature.

[242] Singh V.K., Thakral D., Gupta R. (2021). Regulatory Noncoding RNAs: Potential Biomarkers and Therapeutic Targets in Acute Myeloid Leukemia. Am. J. Blood Res..

[243] Abplanalp W.T., Fischer A., John D., Zeiher A.M., Gosgnach W., Darville H., Montgomery R., Pestano L., Allée G., Paty I. (2020). Efficiency and Target Derepression of Anti-MiR-92a: Results of a First in Human Study. Nucleic Acid Ther..

[244] Chang S., Wu W. (2018). Construction of Multi-Potent MicroRNA Sponge and Its Functional Evaluation. MicroRNA and Cancer.

[245] Jung J., Yeom C., Choi Y.-S., Kim S., Lee E., Park M.J., Kang S.W., Kim S.B., Chang S. (2015). Simultaneous Inhibition of Multiple Oncogenic MiRNAs by a Multi-Potent MicroRNA Sponge. Oncotarget.

[246] Winkle M., El-Daly S.M., Fabbri M., Calin G.A. (2021). Noncoding RNA Therapeutics—Challenges and Potential Solutions. Nat. Rev. Drug Discov..

[247] Hong D.S., Kang Y.-K., Borad M., Sachdev J., Ejadi S., Lim H.Y., Brenner A.J., Park K., Lee J.-L., Kim T.-Y. (2020). Phase 1 Study of MRX34, a Liposomal MiR-34a Mimic, in Patients with Advanced Solid Tumours. Br. J. Cancer.

[248] Van Zandwijk N., Pavlakis N., Kao S.C., Linton A., Boyer M.J., Clarke S., Huynh Y., Chrzanowska A., Fulham M.J., Bailey D.L. (2017). Safety and Activity of MicroRNA-Loaded Minicells in Patients with Recurrent Malignant Pleural Mesothelioma: A First-in-Man, Phase 1, Open-Label, Dose-Escalation Study. Lancet Oncol..

[249] Ochoa S., Milam V.T. (2020). Modified Nucleic Acids: Expanding the Capabilities of Functional Oligonucleotides. Molecules.

[250] Yin L., Liu Y., Peng Y., Peng Y., Yu X., Gao Y., Yuan B., Zhu Q., Cao T., He L. (2018). PARP Inhibitor Veliparib and HDAC Inhibitor SAHA Synergistically Co-Target the UHRF1/BRCA1 DNA Damage Repair Complex in Prostate Cancer Cells. J. Exp. Clin. Cancer Res..

[251] Valdez B.C., Li Y., Murray D., Liu Y., Nieto Y., Champlin R.E., Andersson B.S. (2018). Combination of a Hypomethylating Agent and Inhibitors of PARP and HDAC Traps PARP1 and DNMT1 to Chromatin, Acetylates DNA Repair Proteins, down-Regulates NuRD and Induces Apoptosis in Human Leukemia and Lymphoma Cells. Oncotarget.

[252] Zhao J., Kelnar K., Bader A.G. (2014). In-Depth Analysis Shows Synergy between Erlotinib and MiR-34a. PLoS ONE.

[253] Cortez M.A., Valdecanas D., Niknam S., Peltier H.J., Diao L., Giri U., Komaki R., Calin G.A., Gomez D.R., Chang J.Y. (2015). In Vivo Delivery of MiR-34a Sensitizes Lung Tumors to Radiation Through RAD51 Regulation. Mol. Ther. Nucleic Acids.

